# Comparative analysis of phosphorylated proteomes between plerocercoid and adult *Spirometra mansoni* reveals phosphoproteomic profiles of the medical tapeworm

**DOI:** 10.1186/s13071-024-06454-8

**Published:** 2024-08-31

**Authors:** Yong Yan Liu, Rui Jie Wang, Si Si Ru, Fei Gao, Wei Liu, Xi Zhang

**Affiliations:** 1 Department of Parasitology, School of Basic Medical Sciences, Zhengzhou University, Zhengzhou, 450001 Henan China; 2 Department of Clinical Microbiology, The People’s Hospital of Xixian, Xinyang, 464300 Henan China; 3 Research Center for Parasites & Vectors, College of Veterinary Medicine, Hunan Agricultural University, Changsha, 410128 Hunan China

**Keywords:** *Spirometramansoni*, Plerocercoid, Phosphoproteome, Differentially abundant proteins, 4D label-free quantitative analysis

## Abstract

**Background:**

Plerocercoid larvae of the tapeworm *Spirometra mansoni* can infect both humans and animals, leading to severe parasitic zoonosis worldwide. Despite ongoing research efforts, our understanding of the developmental process of *S. mansoni* remains inadequate. To better characterize posttranslational regulation associated with parasite growth, development, and reproduction, a comparative phosphoproteomic study was conducted on the plerocercoid and adult stages of *S. mansoni*.

**Methods:**

In this study, site-specific phosphoproteomic analysis was conducted via 4D label-free quantitative analysis technology to obtain primary information about the overall phosphorylation status of plerocercoids and adults.

**Results:**

A total of 778 differentially abundant proteins (DAPs) were detected between adults and plerocercoids, of which 704 DAPs were upregulated and only 74 were downregulated. DAPs involved in metabolic activity were upregulated in plerocercoid larvae compared with adults, whereas DAPs associated with binding were upregulated in adults. Gene Ontology (GO) and Kyoto Encyclopedia of Genes (KEGG) analyses indicated that most DAPs involved in signal transduction and environmental information processing pathways were highly active in adults. DAPs upregulated in the plerocercoid group were enriched mainly in metabolic activities. The kinases PKACA, GSK3B, and smMLCK closely interact, suggesting potential active roles in the growth and development of *S. mansoni*.

**Conclusions:**

The dataset presented in this study offers a valuable resource for forthcoming research on signaling pathways as well as new insights into functional studies on the molecular mechanisms of *S. mansoni*.

**Graphical abstract:**

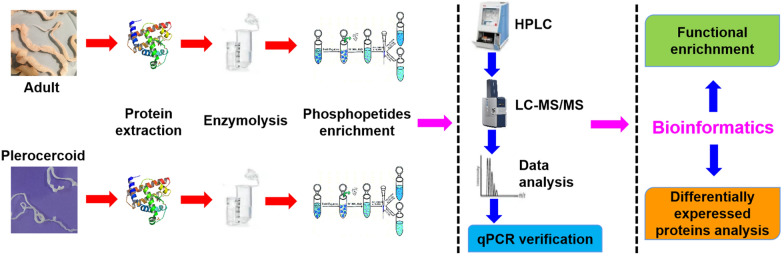

**Supplementary Information:**

The online version contains supplementary material available at 10.1186/s13071-024-06454-8.

## Background

The motile larva (plerocercoid) of *Spirometra mansoni* can invade humans, causing a parasitic zoonosis known as sparganosis [1, 2]. To date, more than 2000 sparganosis cases have been reported, with the majority coming from Asian countries, especially China, Korea, and Japan [3]. The complex interactions between the plerocercoid and human host during infection are the causes of severe pathological changes that can lead to fatal outcomes. A comprehensive understanding of these interactions is crucial for the development of effective intervention strategies [4]. However, despite the long history of the neglected tapeworm, our knowledge of the molecular interactions between this parasite and human hosts remains limited.

Phosphoproteomic techniques are rapidly developing technologies that can provide insights into the interaction mechanisms between pathogens and their hosts [5]. Previous studies have confirmed that the phosphorylation of proteins plays important roles in regulating signaling pathways involved in various interactions between pathogens and hosts [6]. Protein kinases modify the functions of other proteins through the addition of phosphate groups, leading to alterations in the activity, stability, interactions, and localization of targeted proteins [7]. The diverse recognition and modification capabilities of distinct protein kinases towards specific sites on different proteins significantly enhance the intricacy of phosphorylated proteins [6, 7]. Recently, phosphoproteomic techniques utilizing phosphopeptide enrichment methods combined with mass spectrometry (MS) have been employed to investigate large-scale protein phosphorylation profiles in many parasites, such as *Leishmania* spp., *Toxoplasma gondii*, *Schistosoma mansoni*, *Schistosoma mekongi*, and *Echinococcus granulosus* [8–13].

Different life cycle stages of parasites usually encounter different environments; therefore, the transition from one stage to the next generally requires physical and biochemical changes accompanied by altered expression of genes [14]. For *S. mansoni*, understanding the biological process of the transition from the plerocercoid state to the adult state is particularly important because the surrounding environment changes from that of poikilothermic intermediate/paratenic hosts to that of homeothermic definitive hosts to complete their development and reproduction. Therefore, understanding the phosphoproteomic profiles of *S. mansoni* at different developmental stages is critical for exploring the molecular mechanisms of the development and adaptive parasitism of the parasite. With the continuous development of omics technology, some achievements have been made in *Spirometra* tapeworm research at the molecular level. Liu et al. [15] performed a phosphoproteomic analysis to determine the protein phosphorylation networks in *Spirometra erinaceieuropaei* plerocercoid. A comparative study of transcriptomic changes between the adult and larval stages of *S. erinaceieuropaei* was subsequently performed to characterize differential and specific genes and pathways associated with tapeworm development [16]. More recently, integrated transcriptomic and proteomic analyses of *S. mansoni* illustrated three potentially key pathways involved in the development of this medical parasite [17]. These pioneering works provided a solid basis for exploring the phosphoproteomic profiles of *S. mansoni* across different life cycles.

In this study, we performed a comparative phosphoproteome analysis between the adult and plerocercoid stages of *S. mansoni* via 4D label-free analysis technology. This technology has advantages in simple sample processing without labeling, automatic operation, strong separation ability, and wide application range, as well as identifying any type of protein, including membrane, nuclear, and extracellular proteins [18]. More specifically, the following objectives were addressed: (1) performing a comprehensive phosphoproteome analysis between adults and plerocercoids and (2) identifying key proteins associated with the development, reproduction, and survival of *S. mansoni*.

## Methods

### Sample collection

**Fig. 1:**
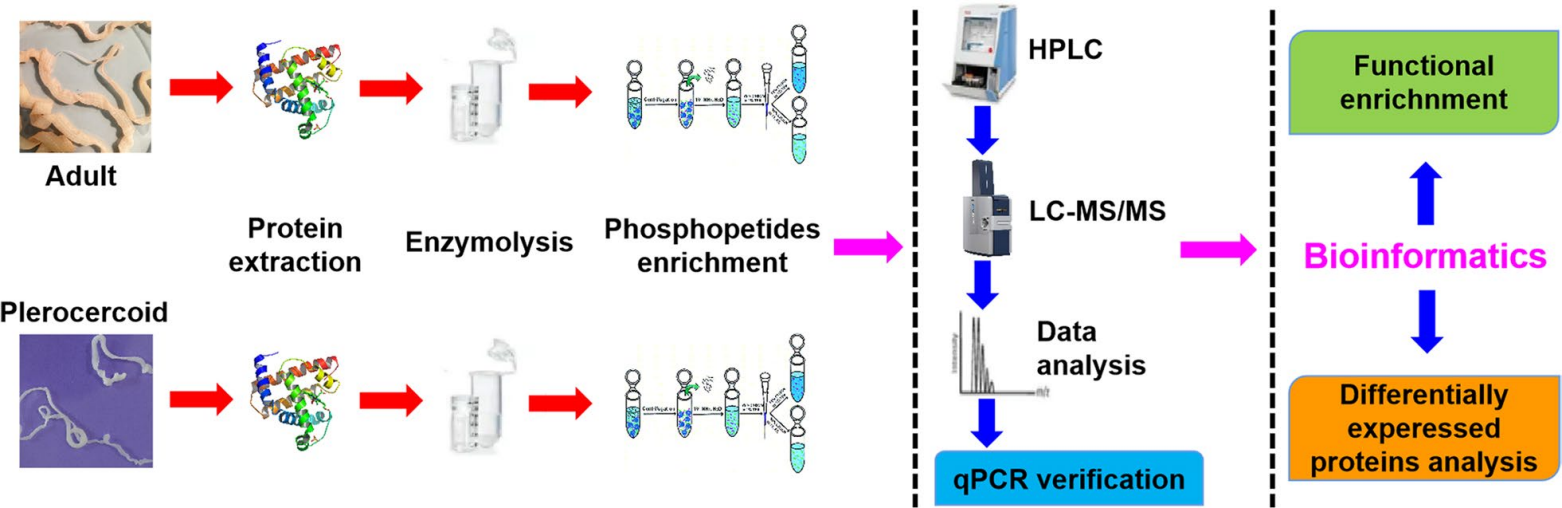
Flow chart of the entire study. Adults and plerocercoids were prepared. Protein extraction, enzymatic hydrolysis, and phosphopeptide enrichment were carried out. Peptide fragments were analyzed by LC‒MS/MS. RT-qPCR quantitative verification and bioinformatics analyses of differentially expressed proteins were performed

The plerocercoids used in this study were isolated from wild snakes and identified as *S. mansoni* through genotyping method described in Kuchta et al. [2]. The head of the plerocercoid was utilized to infect a cat and obtain the adult worm, while the body was subjected to proteomic sequencing. An adult tapeworm was obtained from an infected cat according to procedures described previously [19]. All collected samples were snap-frozen in liquid nitrogen and stored at − 80 °C for further use. A flow chart of the whole study is shown in Fig. 1.

### Extraction and digestion of proteins

Pooled plerocercoid (*n* = 3) and gravid segments of pooled adults (*n* = 3) were used for protein extraction. First, all the frozen samples were ground into powder and transferred into a centrifuge tube. Then, four volumes of lysis buffer containing 8 M urea and 1% protease inhibitor cocktail (Abcam, Cambridge, UK) were added, and the mixture was sonicated three times on ice. To remove the remaining debris, centrifugation was performed at 16,000×*g* at 4 °C for 30 min. Subsequently, 4 µl of the supernatant was used for quantification. The protein concentration was assessed via the BCA method according to the manufacturer’s instructions (Pierce™ BCA Protein Assay Kit, Thermo Fisher Scientific). SDS-PAGE electrophoresis was performed to evaluate whether the concentration protein was appropriate for the subsequent experiments. The trypsin digestion of proteins was performed as described by Ren et al. [20]. In brief, the protein mixture was reduced with 10 mM TCEP at 37 °C for 60 min, followed by alkylation with 40 mM iodoacetamide at room temperature in the dark for 40 min. Precooled acetone (acetone:sample v/v = 6:1) was added to each tube, and the mixture was allowed to react at – 20 °C for 4 h and 10,000×*g* for 20 min. Finally, the sample was completely dissolved with 100 µl of 100 mM TEAB; The solution was digested with trypsin (Promega, Germany) at a ratio of 50:1 (protein/enzyme) at 37 °C overnight.

### Liquid-phase tandem mass spectrometry

The proteins were analyzed by liquid chromatography coupled with tandem mass spectrometry (LC-MS/MS) as described by Capriotti et al. [21]. Briefly, the trypsin peptides dissolved in solvent A (2% acetonitrile, 0.1% formic acid) were directly loaded onto a reverse-phase analysis column (25 cm length, 75 μm internal diameter). Then, solvent B (80% acetonitrile, 0.1% formic acid) was gradually increased as follows: 3% to 28% over 45 min, 28% to 44% over 5 min, and finally up to 90% over 5 min. The peptides were subjected to nanospray ionization (NSI) followed by data-dependent acquisition (DDA) tandem mass spectrometry (MS/MS) in a timsTOF Pro2 (Bruker, Germany) coupled with an Ionopticks UPLC (Ionopticks, USA) system. Full-scan detection of intact peptides was conducted using a 100–1700 m/z scan range and a 1.5 kV electrospray voltage. The data acquisition software used was Compass HyStar (Bruker, Germany). A data-dependent procedure that switched between one MS scan and MS/MS scans was initiated in the MS survey scan with 24 s dynamic exclusion, with a cycle window time of 1.17 s.

### Phosphopeptide identification

MaxQuant v2.0.3.1 (Matrix Science, UK) was used to retrieve mass spectrometry data. Specifically, the retrieval parameter settings were set by comparing transcriptome databases (Bioproject No. PRJNA761840) with reverse library searches to determine the false discovery rate (FDR) resulting from random matching. A common contamination library was compared with the database to eliminate the influence of contaminating proteins. Enzyme digestion was specified as trypsin/P with two missing cut-off points. The precursor mass tolerance was set to 10 ppm. Methionine oxidation, N-terminal acetylation of proteins, and phosphorylation of serine, threonine, and tyrosine were used as alterable modification references with cysteine alkylation as a fixed modification reference. The FDR for protein and peptide-spectrum match (PSM) identification was set to less than 0.01%.

### Bioinformatic analyses

Models of protein sequences in terms of amino acids at particular positions of modified 21-mers (10 amino acids upstream and downstream of the site) in all protein sequences were analyzed via the motif-x program (http://motif-x.med.Harvard.edu/motif-x.html) [22]. Protein sequences in the database were used as background parameters, while the other parameters used default settings. A characteristic sequence was regarded as a motif of the modified peptide if the peptide number in the sequence was > 20 and if the statistical test result was *P* < 0.01. Blast2GO software was used for BLAST analysis [23]. Gene Ontology (GO) is a standardized functional classification system that provides a dynamically updated standardized vocabulary and describes the properties of genes and gene products in organisms in terms of biological process (BP), molecular function (MF), and cell component (CC) [24]. Phosphoprotein functional enrichment analysis was performed via BINGO according to Gene Ontology (GO) [25] and plug-ins in the Cytoscape platform [26]. Significantly enriched phosphoprotein GO terms were compared to those of the group that contained both non-phosphoproteins and phosphoproteins. The hypergeometric distribution of selected phosphoproteins to specific branches in the GO classification was calculated. In the GO analysis, a hypothetical *P* value was returned for each GO term in which phosphoproteins were present, and a small *P* value (*P* < 0.05) indicated enrichment. Fisher’s exact test was used to assess the enrichment of differentially modified proteins among all identified proteins. The InterPro database (http://www.ebi.ac.uk/interpro/) and Fisher’s exact test were used to analyze each type of phosphoprotein. The protein domains were considered significant when the corrected *P* value was < 0.05. The Cluster of Orthologous Groups of proteins (COGs), a protein database established and managed by NCBI, provides information on the phylogenetic categorization of proteins encoded by complete genomes of bacteria, algae, and eukaryotes. The protein sequence was annotated into the COG database by comparison, and each COG cluster was composed of orthologous sequences to predict the function of the sequence. Enriched pathways were identified via a database (https://www.genome.jp/kegg/).

### Kinase and substrate prediction analysis

Protein kinases regulate the activity of substrate proteins, cell localization, and protein interactions by phosphorylating protein substrates. The analysis was performed on the basis of a phosphorylation database (https://www.phosphosite.org/homeAction.action) [27]; the kinase types and the relationships between the substrates were analyzed by means of network graph visualization.

### Screening differentially abundant proteins

According to the differences in protein content between adults and plerocercoids, the protein abundance ratio (that is, the difference multiple) was > 1.2, and the *P* value was < 0.05. These proteins were regarded as differentially abundant proteins (DAPs) between different samples. GO annotation was subsequently performed for the DAPs. The adult group was used as the control, and a histogram of up- and downregulated protein GO annotations was generated. GO functional enrichment analysis was performed to determine the functional enrichment of the DAPs and clarify the differences between the samples at the functional level. The same method was used as described above. The KEGG database was used to classify proteins according to their pathways or functions. The differential proteins grouped into pairs are shown in the KEGG pathway diagram, with the adult group serving as the control group. The analysis method was the same as that described above. The corrected *P* value (FDR) was set at a threshold of 0.05. In this analysis, *P* < 0.05 according to Fisher’s exact test was taken as the threshold, and a KEGG pathway meeting this criterion was considered to be significantly enriched in DAPs.

### Interaction analysis of the DAPs

The differential protein interaction network was analyzed using the STRING database (http://string-db.org/). The STRING database searches for interactions between known and predicted proteins [28]. Visual analysis of the metabolic pathways of the DAPs was performed via iPath3.0 (http://pathways.embl.de) to obtain the metabolic pathway information of the entire biological system.

### Experimental corroboration using qRT-PCR

A total of 16 DAPs (8 upregulated proteins and 8 downregulated proteins) in each plerocercoid and adult were randomly selected for analysis to validate the proteomic data. Total RNA was extracted using TRIzol reagent (Invitrogen, USA) according to the manufacturer’s instructions. The RNA was first dissolved in RNase-free ddH_2_O (Takara, China), after which cDNA was synthesized via a reverse transcription kit (Novoprotein, Shanghai, China). The reverse transcribed first-strand cDNA was used as a template. The primers used for RT-PCR were synthesized by Sangon Biotech (Shanghai, China) (Supplementary Table S5). qPCR (quantitative PCR) was performed on an Applied Biosystems 7500 Fast Real Time PCR System (Applied Biosystems, USA). GAPDH was used as an endogenous control gene. Statistical differences between two groups were analyzed using Student’s t-test with significant differences at *P* < 0.05.

### Data deposition

All the data supporting the findings of this article are included in the main article and its supplementary files. The mass spectrometry proteomics data have been deposited in the ProteomeXchange Consortium (http://proteomecentral.proteomexchange.org) via the iProX partner repository [29] with the dataset identified as PXD048134.

## Results

### Phosphoproteome of *S. mansoni*

**Fig. 2:**
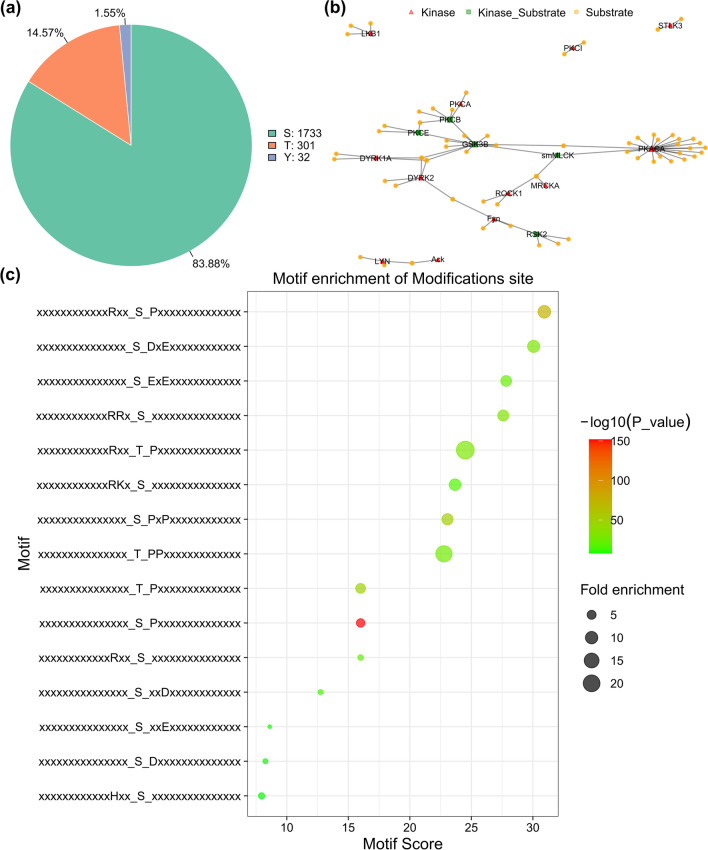
Phosphoproteomic analysis of the different developmental stages of *Spirometra mansoni* via 4D label-free quantitative analysis. **a** Pie chart representation of the distribution of identified phosphorylation sites. Note: Green indicates phosphoserine, orange indicates phosphothreonine, and blue indicates phosphotyrosine. **b** Network diagram of kinases and corresponding substrates. For convenience of visualization, only nodes with centrality in the top 50 degrees are displayed. Note: Red triangles represent kinases, green squares indicate that the gene product can be either a kinase or a substrate, orange circles indicate that the gene product is a substrate, and lines indicate that there is a kinase-substrate interaction. **c** Enriched bubble patterns of identified motif sequences. Note: In the figure, the abscissa represents the motif score Motif_score, and the ordinate represents various motif sequences, which are sorted by motif score. The bubble size indicates the enrichment factor, and the larger the bubble is, the greater the fold enrichment. The bubble color indicates the − log10 (*P* value). The smaller the *P* value is, the more significant the difference. The results are shown in red

In this study, we report the first global phosphoproteomic dataset of the different developmental stages of *S. mansoni*. In total, 1731 phosphopeptides and 2066 phosphorylation sites were identified. Details on the identified phosphopeptides are provided in Supplementary Table S1. Among the 1731 phosphopeptides, the length of the peptides ranged from 7 to 39 amino acids, with most being 12–21 amino acids (Supplementary Fig. S1). Among the 2066 phosphorylation sites, phosphoserine comprised the greatest proportion (83.9%), followed by phosphothreonine (14.6%) and phosphotyrosine (1.5%) (Fig. 2a). In addition, 76.5% (1581/2066) of the phosphorylated peptides were located at the first site of the peptide, 9.1% (188/2066) were located at the second site, and 8.5% (176/2066) were phosphorylated at both sites (Supplementary Fig. S2). BLAST analysis found that 61.6% (726/1179) of the identified phosphoproteins were annotated with at least one GO node in each of the three GO categories (molecular function, cellular component, and biological process). The distribution of the three categories of GO terms for the *S. mansoni* phosphoproteins is shown in Supplementary Fig. S3 and Supplementary Table S2. The COG annotation results showed that 93 proteins were involved in 25 functional classifications (Supplementary Fig. S4). KEGG analysis revealed that 562 proteins were classified into six categories (metabolism, genetic information processing, environmental information processing, cellular processes, organismal systems, and human disease) that mapped to 295 pathways (Supplementary Fig. S5). Moreover, the types of kinases involved were determined, and the relationships between kinases and substrates were visualized through a network graph (Fig. 2b). The kinase PKACA is associated with a variety of phosphorylated protein substrates.

The occurrence of each amino acid in phospho-21-mers was compared to determine the composition of the amino acid residues flanking the phosphorylation sites. Phosphosites were detected from the motif-x algorithm, with 15 motifs categorized as a result. The motifs identified included 12 phosphoserine motifs and 3 phosphothreonine motifs. The phosphotyrosine motifs were undetectable, possibly because of the low abundance of tyrosine-phosphorylated peptides. Phosphoserine motifs containing “…R.SP…” (fold increase = 10.5), “…SD.E…” (fold increase = 9.5), and “…RK S…” (fold increase = 8.5) were the top three motifs phosphorylated by kinases (Fig. 2c). The three phosphothreonine motifs were “…R.TP…” (fold increase = 22.9), “…TPP…” (fold increase = 18.7), and “…TP…” (fold increase = 6).

### Screening of differentially abundant proteins

**Fig. 3:**
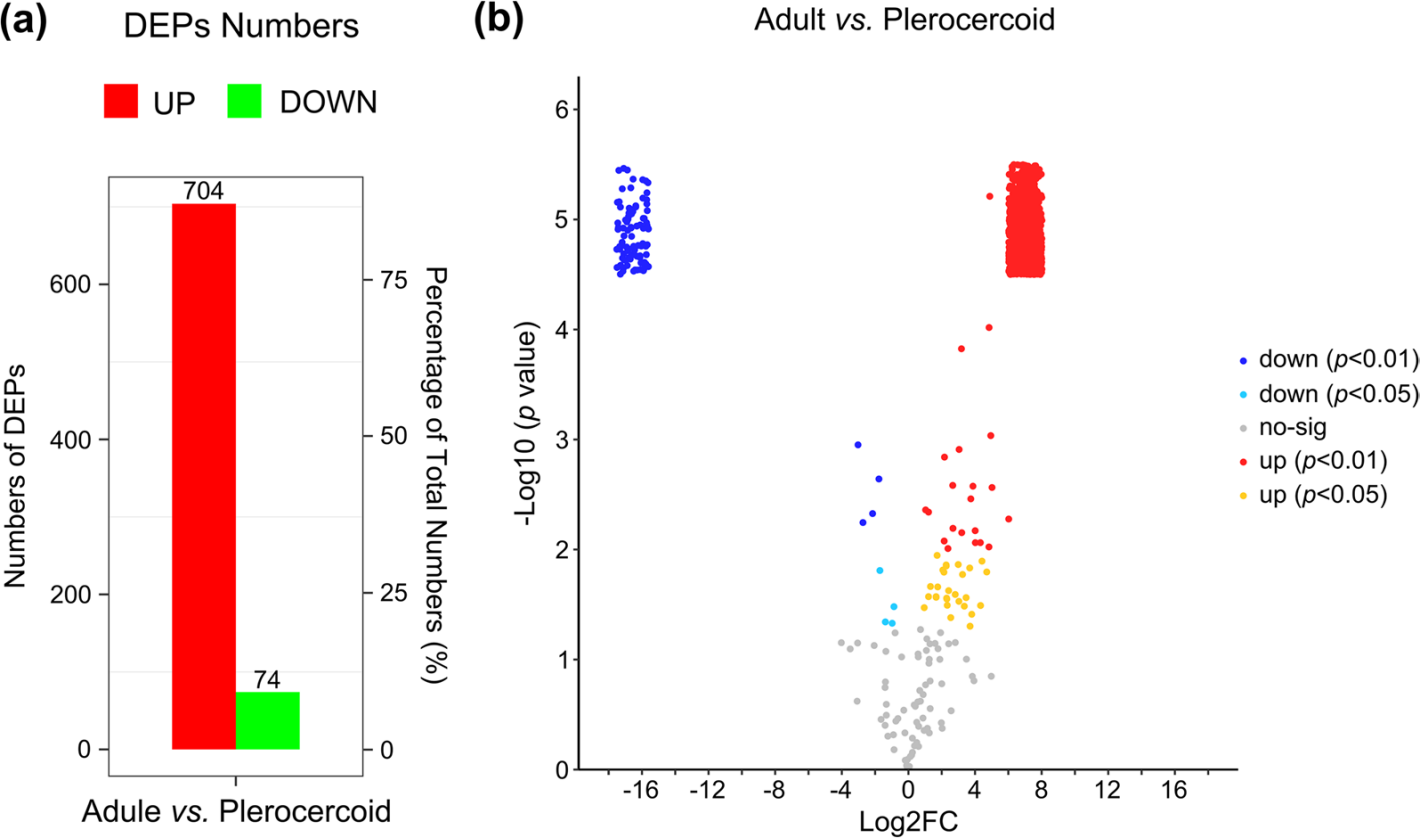
Differentially abundant protein (DAP) profiles were determined via 4D label-free quantitative analysis. **a** Quantitative distribution of DAPs between the adult and plerocercoid stages. Red indicates significant upregulation, and green indicates significant downregulation. **b** Visual volcano map of different peptide segments in each subgroup. Note: The horizontal coordinate is the multiple change in the difference between the two samples. The ordinate is the statistical t-test *P* value of the difference in peptide expression, and the smaller the value, the more significant the difference in expression. Yellow indicates peptides that were significantly upregulated with *P* < 0.05, red indicates peptides that were significantly upregulated with *P* < 0.01, light blue indicates peptides that were significantly downregulated with *P* < 0.05, blue indicates peptides that were significantly downregulated with *P* < 0.01, and black indicates peptides that were not significantly differentially expressed

Volcano plots were generated to visualize proteins that were potentially responsible for the difference between adults and plerocercoids (*P* < 0.05 and > 1.5 or < 0.66-fold change) (Fig. 3a). A total of 778 proteins were identified as DAPs between adults and plerocercoids, among which 704 and 74 proteins were up- and downregulated, respectively (Fig. 3b). The DAPs in the two developmental stages are listed in Supplementary Table S3.

### Enrichment analysis of DAPs

**Fig. 4:**
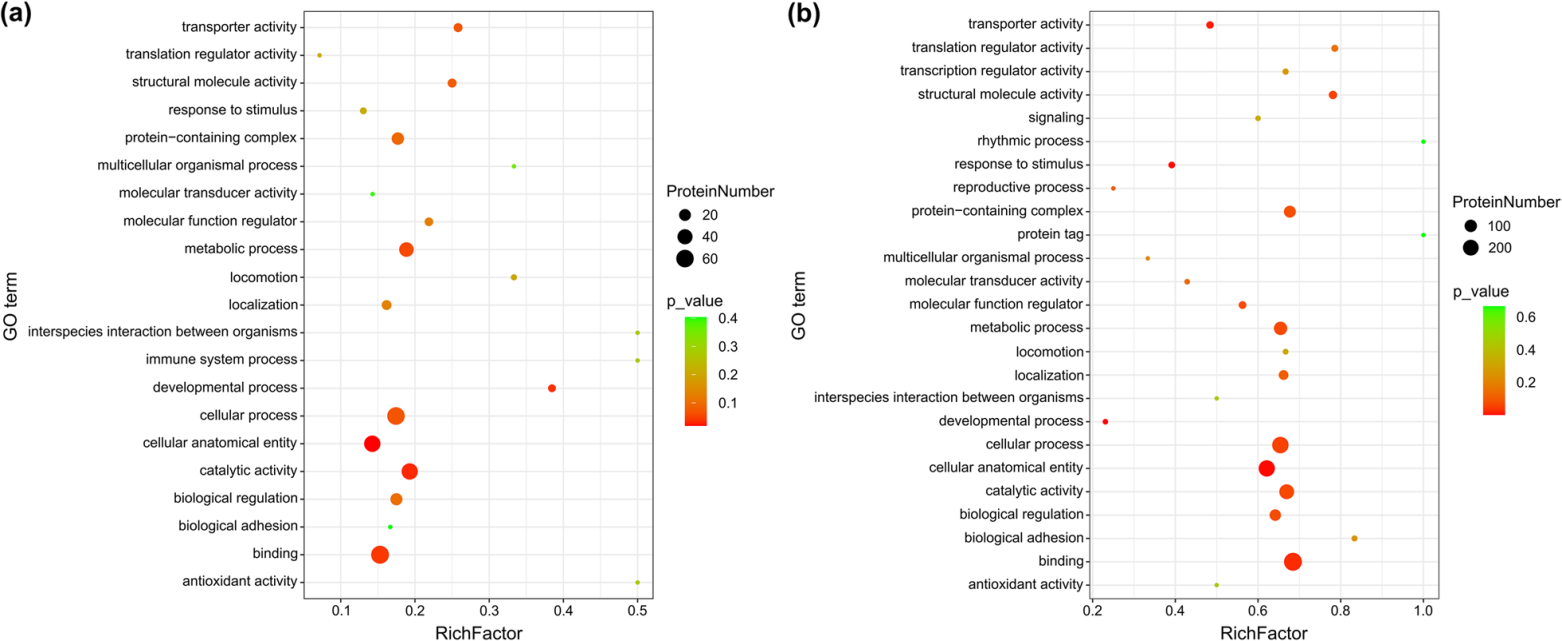
GO functional enrichment of the differentially abundant proteins. **a** Enrichment of significantly differentially expressed proteins in plerocercoids. **b** Enrichment of significantly differentially abundant proteins in adults. Each bubble represents a GO term. The color of the bubble indicates the significance of enrichment. The size of the bubble refers to the quantity of enriched proteins in the respective pathway

GO enrichment analysis was performed to identify processes enriched in the DAPs. Among the 778 DAPs between adults and plerocercoids, 493 were successfully annotated with 26 GO terms (14 biological processes, 10 molecular functions, and 2 cell components) (Supplementary Fig. S6). To obtain a detailed view of stage-specific genes, GO term enrichment analyses were also performed to assess significantly overrepresented GO terms. At the plerocercoid stage, 121 DAPs were classified into 21 GO terms. The top three GO subclasses enriched were cationic binding, anion binding, and nucleoside phosphate binding (Fig. 4a). For the adult stage, 482 DAPs were classified into 25 GO terms. The most enriched GO subclasses included anion binding, intracellular organelles, nucleoside phosphate binding, nucleotide binding, and nucleic acid binding (Fig. 4b).

**Fig. 5:**
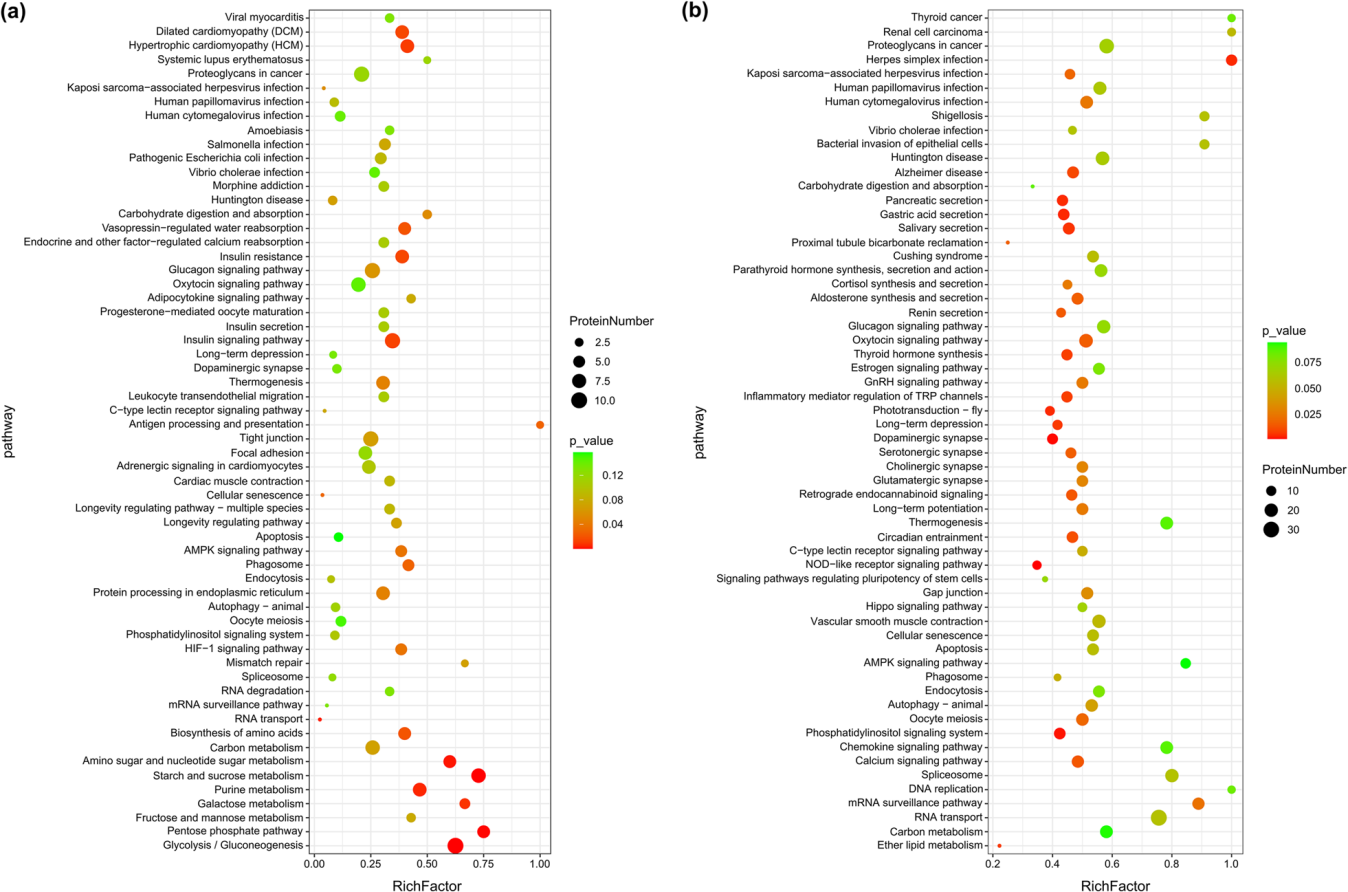
KEGG enrichment analysis of differentially abundant proteins in adults vs. plerocercoids. **a** Enrichment analysis of the most significant KEGG pathways of upregulated proteins in plerocercoids. **b** Enrichment analysis of the most significant KEGG pathways associated with the upregulated proteins in adults. Each bubble in the visualization represents a specific pathway, with the size of the bubble indicating the abundance of enriched proteins associated with that pathway. The y-axis labels provide the name of the corresponding pathway

To explore the changes in biological pathways operating during the two stages, differentiated proteins were mapped to reference pathways in the KEGG database. The findings implied that 372 DAPs matched 280 KEGG pathways in the adult stage and that 94 DAPs matched 187 KEGG pathways in the plerocercoid stage (Supplementary Fig. S7). Moreover, KEGG enrichment analysis was performed to assess significantly overrepresented KEGG terms to obtain a detailed view of stage-specific upregulated proteins. In the plerocercoid stage, glycolysis/gluconeogenesis, proteoglycans in cancer, tight junctions, the glucagon signaling pathway, and the insulin signaling pathway were the most highly enriched KEGG pathways (Fig. 5a). In the adult stage, the top enriched pathways were RNA transport, cancer pathways, proteoglycans in cancer, tight junctions, and platelet activation (Fig. 5b). Moreover, a representative pathway map of RNA transport is presented in Fig. 6. A total of 31 important DAPs, such as nuclear pore protein, RNA helicase, eukaryotic translation initiation Factor 3 subunit G, and small ubiquitin-related modifier, were found in this pathway. Among these DAPs, 30 proteins were upregulated in adults, and only 1 protein was upregulated in plerocercoids, indicating that the RNA transport pathway is more active in adults.

**﻿Fig. 6:**
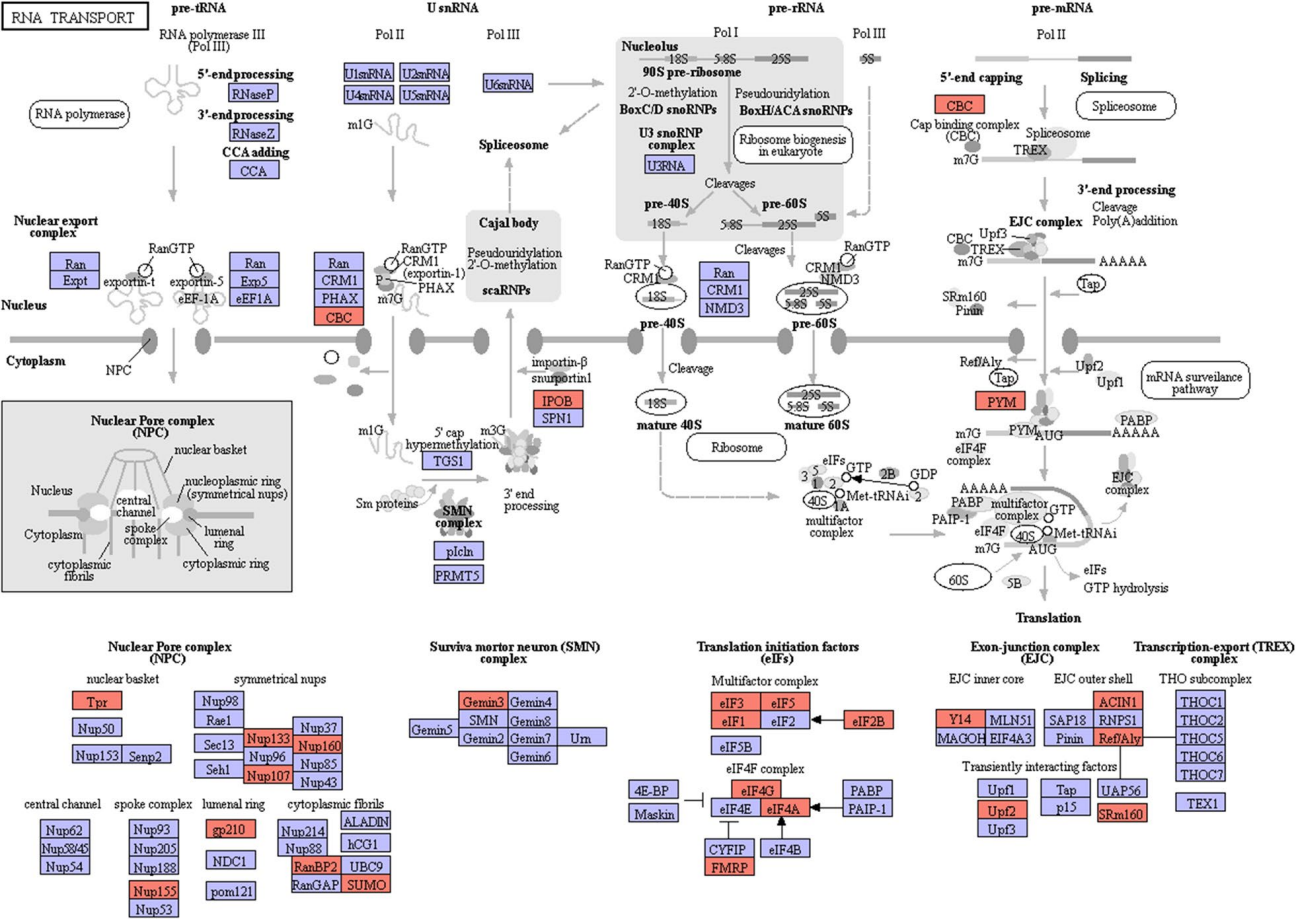
Representative KEGG pathway associated with RNA transport. Thirty-one differentially abundant proteins between adults and plerocercoids were involved in the RNA transport pathway. Red and green symbols represent proteins whose expression was up- or downregulated in adults, respectively

### PPI analysis of DAPs

**Fig. 7:**
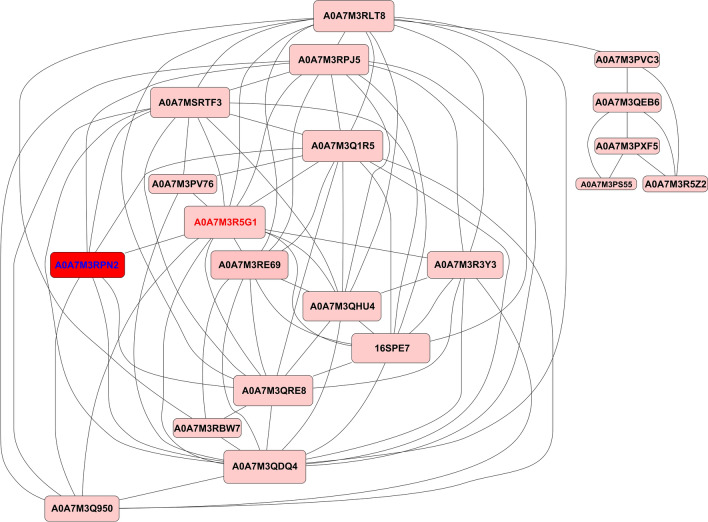
Protein-protein interaction network analysis. The nodes in the figure represent differentially abundant proteins, with the downregulated proteins noted in green and the upregulated proteins noted in red

A protein-protein interaction network was constructed to assess the relationships among the DAPs. Using the evaluation index of connectivity degree in combination with related literature analysis, 30 proteins with high connectivity were selected as key proteins in the PPI analysis (Fig. 7). Among these DAPs, A0A7M3RPN2 (60S ribosomal protein L6) was the most prominent protein. A0A7M3RPN2 interacts with more than seven proteins: A0A7M3Q1R5 (40S ribosomal protein S3a), A0A7M3R5G1 (40S ribosomal protein S6), A0A7M3QDQ4 (40S ribosomal protein S18), A0A7M3Q950 (60S ribosomal protein L29), A0A7M3RTF3 (40S ribosomal protein S12), A0A7M3QRE8 (ribosomal protein), and A0A7M3RPJ5 (40S ribosomal protein S9). Interestingly, all of the above DAPs participate in the genetic information processing pathway (ko03010). A0A7M3R5G1 (40S ribosomal protein S6) is involved in other pathways, such as human disease (ko05205, ko01521), environmental information processing (ko04150, ko04151, ko04066), and organic systems (ko04714, ko04910) (Supplementary Table S4).

### iPath network

**Fig. 8:**
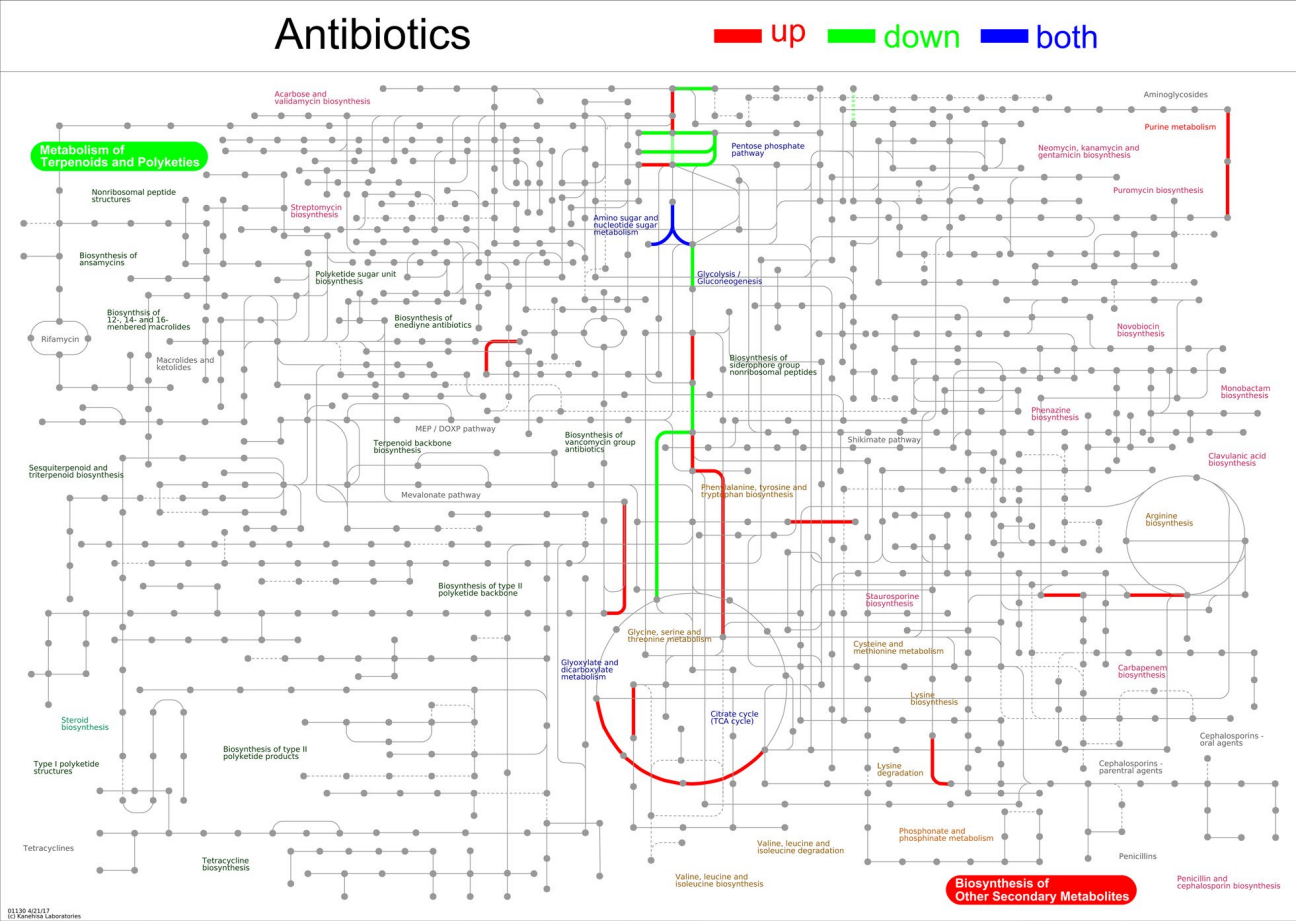
iPath integration path diagram. Differentially abundant proteins associated with antibiotic metabolic pathways in adults vs. plerocercoids. Note: The nodes in the figure represent different compounds, and the boundaries represent different enzymatic reactions. The red lines represent reactions involving upregulated proteins, green lines represent reactions involving downregulated proteins, and the blue lines represent reactions involving both up- and downregulated proteins. Different lines represent different types of metabolic pathways or functions

To view the metabolic pathway information of the entire biological system and outline the biosynthesis and important regulatory pathways of secondary metabolites, a visual analysis of metabolic pathways was performed. In the antibiotic metabolic pathway, the upregulated DAPs in adults were mainly involved in the biosynthesis of other secondary metabolites, including phenylalanine, tyrosine, and tryptophan; purine metabolism; and other pathways. The downregulated DAPs were primarily implicated in terpenoid and polyketone metabolic pathways. Both up- and downregulated proteins participated in the metabolic reactions of amino sugars and nucleotide sugars (Fig. 8). In the metabolic pathway, the upregulated DAPs were mostly involved in the metabolism of coenzyme factors and vitamins, whereas the downregulated DAPs were predominantly involved in the metabolism of terpenoids and polyketones (Supplementary Fig. S8). In the microbial metabolic pathway, the upregulated DAPs were mainly involved in the degradation of chloroalkanes, the C4-dicarboxylic acid cycle, and light and carbon fixation in organisms. The downregulated DAPs were mainly associated with phosphate metabolism and styrene degradation (Supplementary Fig. S9). In the secondary metabolic pathway, the upregulated DAPs were mainly involved in the metabolic pathways of coenzyme factors and vitamins. The downregulated DAPs were mostly associated with the terpenoid and polyketone metabolic pathways (Supplementary Fig. S10).

### Experimental corroboration of proteomic data

**Fig. 9:**
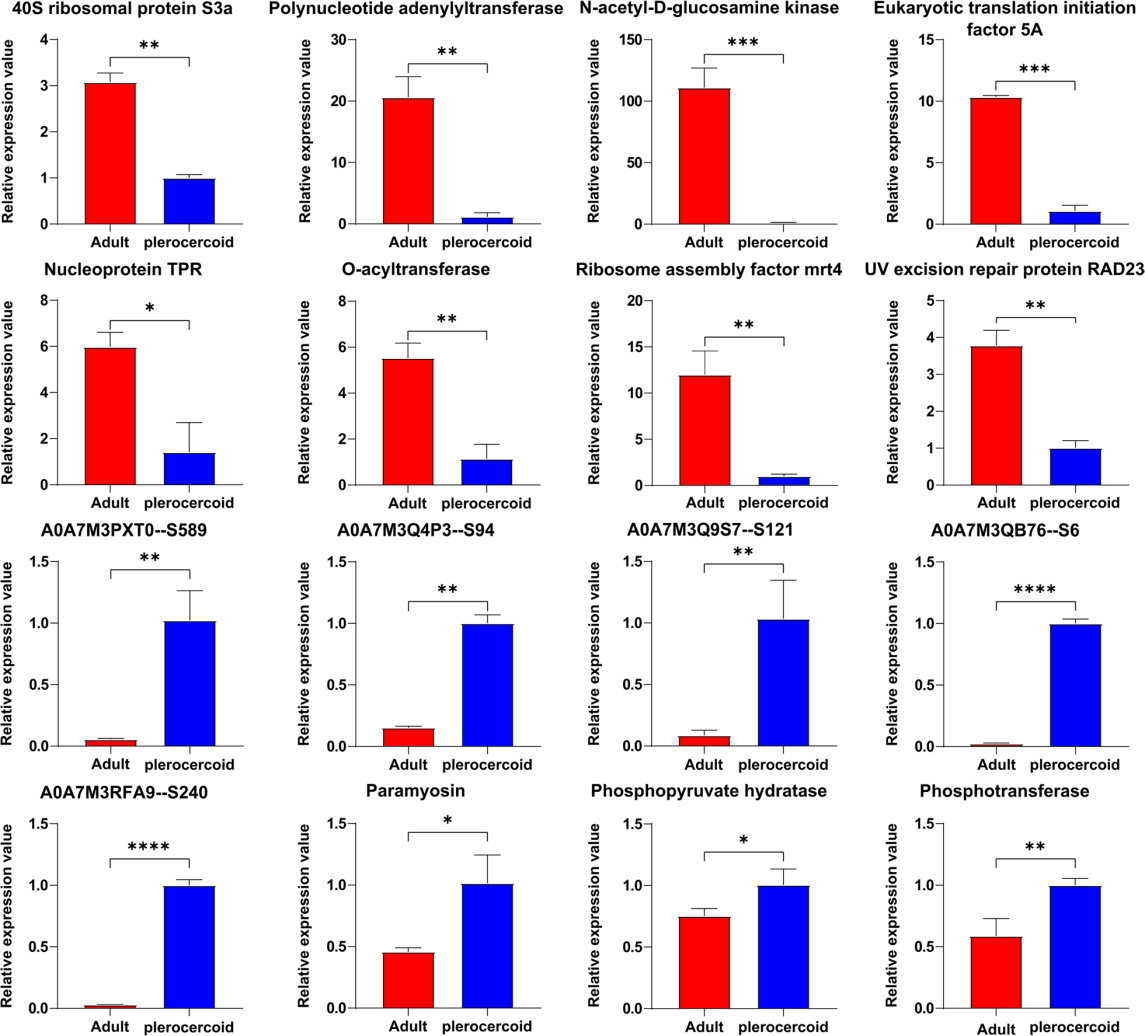
RT‒qPCR validation of differentially expressed *Spirometra mansoni* genes. GAPDH was used for normalization. The results are presented as the mean ± SEM (standard mean of error) of samples (*n* = 3). Asterisks indicate significant differences (^*^P < 0.05, ^**^P < 0.01, ^***^P < 0.001, ^****^P < 0.0001)

The expression levels of 16 randomly selected DAPs were calculated via the 2^−ΔΔCt^ method. According to the sequencing results, the expression levels of 40S ribosomal protein S3a (A0A7M3Q1R5), polynucleotide adenylyltransferase (A0A7M3PRQ6), *N*-acetyl-d-glucosamine kinase (A0A7M3QF48), eukaryotic translation initiation Factor 5A (A0A7M3QUY0), nucleoprotein TPR (A0A7M3PRG3), *O*-acyltransferase (A0A7M3PSR3), ribosome assembly factor mrt4 (A0A7M3PS07), and the UV excision repair protein RAD23 (A0A7M3PS32) were upregulated in adults. The expression levels of paramyosin (A0A7M3Q648), phosphotransferase (A0A7M3QVA0), phosphopyruvate hydratase (A0A7M3PSD4), hypothetical protein (A0A7M3PXT0), hypothetical protein (A0A7M3Q4P3), hypothetical protein (A0A7M3Q9S7), hypothetical protein (A0A7M3QB76), and hypothetical protein (A0A7M3RFA9) were upregulated in plerocercoids. Accordingly, the expression trends of the selected proteins were consistent with those obtained via proteomics (Fig. 9), confirming the accuracy and reliability of the proteomic results.

## Discussion

The development of a parasitic infection in a host occurs via signals exchanged between the host and the parasite [30]. Posttranslational modifications, such as protein phosphorylation, play important roles in the transmission of these signals, thereby playing a critical role in the process of host infection [31]. It has been confirmed that the phosphorylation of important amino acid sites can lead to the modification of phosphorylated proteins and then have serious effects on the physiological state of the host, allowing for parasite growth and invasion, as well as altering signaling pathways [32]. Therefore, in-depth research on protein phosphorylation is highly important for improving our understanding of the role of protein phosphorylation in parasite growth and development.

A total of 1731 phosphopeptides, 2066 phosphorylation sites, and 1179 proteins were identified in the phosphoproteomes of the adult and plerocercoid stages. The modification rates of phosphoserine, phosphothreonine, and phosphotyrosine in *S. mansoni* are similar to those previously described [33]. Kinase and substrate prediction analyses revealed that the kinase PKACA closely interacts with GSK3B and smMLCK and that GSK3B- and smMLCK-related biological processes can be further regulated by phosphorylating protein substrates. GSK3B, a serine threonine kinase belonging to the glycogen synthase kinase subfamily, is a master regulator of a variety of cellular pathways, including insulin signaling and glycogen synthesis, neurotrophic factor signaling, Wnt signaling, neurotransmitter signaling, and microtubule dynamics [34]. MLCK is the first serine/threonine-specific calmodulin (CaM)-dependent protein kinase discovered to dynamically regulate actomyosin recombination and cell contraction in eukaryotic muscle cells as well as nonmuscle cells in mammals. Simard et al. [35] reported that MLCK not only regulates cell contraction but also regulates cell migration, movement, and apoptosis.

In addition, a total of 778 DAPs were identified between adults and plerocercoids. In a previous transcriptomic study, DEGs enriched in the plerocercoid stage were associated with parasite immune evasion, whereas more upregulated DEGs were associated with metabolic activity in the adult stage [16]. However, few proteins involved in defense mechanisms were detected in this study, and the proteins identified were mostly functional proteins that maintain the normal physiological requirements of the parasite body. Interestingly, among those DAPs, the number of proteins with titin kinase activity was the greatest in this study. Titin, in addition to actin and myosin, is the most abundant protein in skeletal muscle fibers and maintains the stability of adjacent muscle segments by allocating myosin [36]. Myosin regulates active muscle contraction and acts with myosin and actin to precisely regulate coarse and fine muscle filaments to increase muscle contraction [37]. Therefore, we hypothesized that the kinase may play important roles in the movement of *S. mansoni* and invasion of host cells.

Further GO analysis revealed that DAPs were significantly enriched in the functional classification of cellular processes, metabolic processes, and biological regulation. KEGG analysis revealed that most of the DAPs were enriched in pathways related to biological systems. Most of the biological processes involved are related to cytoskeleton regulation, biological transformation, and molecular function regulation and are involved in basic pathways such as RNA transport, the oxytocin signaling pathway, vascular smooth muscle contraction, and platelet activation. Liu et al. [15] reported that the phosphatidylinositol signaling system (environmental information processing; signal transduction) is the most abundant protein phosphorylation pathway. In this study, we found that genes related to signal transduction in the environmental information processing category (the phosphatidylinositol signaling system, cGMP-PKG signaling pathway, and calcium signaling pathway) were significantly enriched. Phosphatidylinositol (PtdIn) is a small lipid molecule that is present on the cytoplasmic surface of cell membranes and plays an important role in vesicle transport and cell localization [38]. cGMP is a ubiquitous cellular second messenger produced by guanylate cyclase (GC), which acts on guanosine triphosphate (GTP) [39]. cGMP has three main targets: cGMP-dependent protein kinase (PKG), cGMP-regulated phosphodiesterase, and cyclic guanosine phosphate gated ion channels [40]. PKG is considered to be the most important downstream target of cGMP [41]. Animal PKG is involved in the regulation of central nervous system physiological activities in the cGMP signal transduction pathway, such as affecting the sensitivity of animals to food odor, smell, and taste, thus influencing the inclination and choice of food [42–44]. There have been many studies on the cGMP-PKG signaling pathway in human-related diseases [45–47], and the pathophysiological processes involved in the life activities of *S. mansoni* require further study. As an important intracellular second messenger, Ca^2+^ regulates the release of transmitters by transmitting information to intracellular systems to regulate vasomotor activity, gene transcription, protein synthesis and decomposition, cell growth, proliferation, apoptosis, and other life processes [48]. When the calcium ions in the body are disturbed, the cells in the body may become severely dysfunctional. The Ca^2+^ signaling pathway plays an important role in the pathogenic process of fungi [49], but little is known about the role of the Ca^2+^ signaling pathway in the related life activities of *S. mansoni*.

Through protein-protein interaction (PPI) network analysis, we found that the protein 60S ribosomal protein L6 (RPL6) was the most prominent protein, indicating that this protein might be more significant than other proteins [50, 51]. RPL6 is an internal protein highly conserved across parasite isolates from a broad geographical distribution. 60S ribosomal protein L6 (RPL6), a novel intermediary molecule connecting three hubs of the metalloprotein network, is a key molecule that regulates zinc- and magnesium-bound metalloproteins in Parkinson’s disease [52]. Bai et al. reported that RPL6 can be transferred from the nucleolus to the nucleoplasm under ribosomal stress, where it promotes HDM2-mediated RPL6 polyubiquitination and proteasome degradation [53]. RPL6 has also been associated with Noonan syndrome, an autosomal dominant developmental disorder [54]. Moreover, RPL6 is highly conserved across global isolates of *Plasmodium falciparum* and is an ideal candidate for subunit vaccination against malaria [55]. Therefore, the function of the RPL6 protein in *S. mansoni* warrants further study. Ribosomal proteins are essential components of ribosomes, the cellular machinery responsible for protein synthesis [56]. To further understand the role of the phosphorylation of ribosomal proteins, KEGG enrichment analysis was performed. Two key proteins, U3 small nucleolar RNA-associated protein 15 homologue (PhosphoSite: S513) and ribosome maturation protein SBDS (PhosphoSite: S248), participate in the eukaryotic ribosomal biogenetic pathway as well as the ribosomal pathway. Homologs of U3 small nuclear RNA-associated protein 15 are crucial in disease development and may serve as a new targets for molecular targeted therapy in the future [57]. SBDS is a crucial ribosomal maturation factor necessary for releasing eukaryotic translation initiation Factor 6 (eIF6) from 60S ribosomal subunits in the final stages of maturation. This release is essential for enabling interactions between the 60S and 40S subunits to form functional 80S monosomes. SBDS plays important roles in the development of hepatocellular carcinoma [58]. Despite the crucial roles of these ribosomal proteins in human disease processes, their characteristics and functions in tapeworms remain poorly understood. Further exploration is thus needed to determine the specific functions of these ribosomal proteins in *S. mansoni*.

## Conclusions

In this study, the 4D label-free technique was used to compare the phosphoproteomes of *S. mansoni* at the adult and the plerocercoid stages for the first time. The differentially abundant proteins in adults were mainly related to environmental information processing pathways, whereas those in plerocercoids were primarily associated with metabolic pathways. The kinases PKACA, GSK3B, and smMLCK interact closely with each other, suggesting that these kinases may play important roles in the life cycle of *S. mansoni*. This study provides a foundation for revealing the growth and development mechanism of *S. mansoni*. In future studies, it will be necessary to further explore the genes closely related to growth and development to clarify the developmental regulatory mechanism of *S. mansoni*.

### Supplementary Information

Supplementary Material 1. **Table S1. **Modification of peptide information.

Supplementary Material 2. **Table S2.** The distribution of the three categories of GO terms for the *Spirometra mansoni* phosphoproteins.

Supplementary Material 3. **Table S3.** The differentially abundant proteins in adult vs. plerocercoid.

Supplementary Material 4. **Table S4.** Protein-protein interaction network analysis*.*

Supplementary Material 5. **Table S5**. Primer sequences designed for RT-qPCR. **Figure S1.** Peptide length of all identified proteins. **Figure S2.** Pie chart of the number distribution of phosphorylation sites on identified phosphorylated peptide segments. **Figure S3.** GO secondary classification chart. **Figure S4.** Functional classification of COG. **Figure S5.** KEGG annotation statistics. **Figure S6.** Differential protein GO annotation bar chart. **Figure S7.** KEGG enrichment bubble chart. **Figure S8.** Ipath integration path diagram of metabolic pathway map. **Figure S9.** Ipath integration path diagram of microbial metabolic pathway map. **Figure S10. **Ipath integration path diagram of secondary metabolic pathway map.

## Data Availability

No datasets were generated or analyzed during the current study.

## Abbreviations

DAP: Differentially abundant protein
MS: Mass spectrometry
LC-MS/MS: Liquid chromatography coupled with tandem mass spectrometry
FDR: False discovery rate
GO: Gene Ontology
KEGG: Kyoto Encyclopedia of Genes and Genomes
COG: Clusters of Orthologous Groups
BP: Biological process
CC: Cellular component
MF: Molecular function
PPI: Protein–protein interaction
RPL6: 60S ribosomal protein L6
qRT-PCR: Quantitative real-time PCR
qPCR: Quantitative PCR
GAPDH: Glyceraldehyde-3-phosphate dehydrogenase

## References

[1] Xu FF, Chen WQ, Liu W, Liu SS, Wang YX, Chen J, et al. Genetic structure of *Spirometra mansoni* (Cestoda: Diphyllobothriidae) populations in China revealed by a Target SSR-seq method. Parasit Vectors. 2022;15:485. 36564786 10.1186/s13071-022-05568-1 PMC9789593

[2] Kuchta R, Kolodziej-sobocinska M, Brabec J, Młocicki D, Sałamatin R, Scholz T. Sparganosis (*Spirometra*) in Europe in the molecular era. Clin Infect Dis. 2021;72:882–90. 32702118 10.1093/cid/ciaa1036

[3] Liu Q, Li MW, Wang ZD, Zhao GH, Zhu XQ. Human sparganosis, a neglected food borne zoonosis. Lancet Infect Dis. 2015;15:1226–35. 26364132 10.1016/S1473-3099(15)00133-4

[4] Chen WQ, Liu SS, Cheng C, Cui J, Wang ZQ, Zhang X. Molecular characteristics of glutathione transferase gene family in a neglect medical *Spirometra* tapeworm. Front Vet Sci. 2022;9:1035767. 36406076 10.3389/fvets.2022.1035767 PMC9666886

[5] Ryan DJ, Spranggins JM, Caprioli RM. Protein identification strategies in MALDI imaging mass spectrometry: a brief review. Curr Opin Chem Biol. 2019;48:64–72. 30476689 10.1016/j.cbpa.2018.10.023 PMC6382520

[6] Wang YC, Peterson SE, Loring JF. Protein post-translational modifcations and regulation of pluripotency in human stem cells. Cell Res. 2013;24:143. 24217768 10.1038/cr.2013.151 PMC3915910

[7] Huang PH, White FM. Phosphoproteomics: unraveling the signaling web. Mol Cell. 2008;31:777–81. 18922462 10.1016/j.molcel.2008.09.001 PMC2754874

[8] Tsigankov P, Gherardini PF, Helmer-Citterich M, Spath GF, Zilberstein D. Phosphoproteomic analysis of diferentiating *Leishmania* parasites reveals a unique stage-specifc phosphorylation motif. J Proteome Res. 2013;12:3405–12. 23688256 10.1021/pr4002492

[9] Simanon N, Adisakwattana P, Thiangtrongjit T, Limpanont Y, Chusongsang P, Chusongsang Y, et al. Phosphoproteomics analysis of male and female *Schistosoma mekongi* adult worms. Sci Rep. 2019;9:10012. 31292487 10.1038/s41598-019-46456-6 PMC6620315

[10] Hirst NL, Nebel J, Lawton SP, Walker AJ. Deep phosphoproteome analysis of *Schistosoma mansoni* leads development of a kinomic array that highlights sex-biased differences in adult worm protein phosphorylation. PLoS Negl Trop Dis. 2020;14:0008115. 10.1371/journal.pntd.0008115 PMC7089424 32203512

[11] Bansal P, Antil N, Kumar M, Yamaryo-Botté Y, Rawat RS, Pinto S, et al. Protein kinase TgCDPK7 regulates vesicular trafficking and phospholipid synthesis in *Toxoplasma gondii*. PLoS Pathog. 2021;17:1009325. 10.1371/journal.ppat.1009325 PMC7909640 33635921

[12] Maurya R, Tripathi A, Kumar M, Antil N, Yamaryo-Botté Y, Kumar P, et al. PI4-kinase and PfCDPK7 signaling regulate phospholipid biosynthesis in *Plasmodium falciparum*. EMBO Rep. 2022;23:e54022. 34866326 10.15252/embr.202154022 PMC8811644

[13] Miles S, Magnone J, García-Luna J, Dematteis S, Mourglia-Ettlin G. Unraveling post-translational modifications in *Echinococcus granulosus* sensu lato. Acta Trop. 2022;230:106410. 35300939 10.1016/j.actatropica.2022.106410

[14] Heizer E, Zarlenga DS, Rosa B, Gao X, Gasser RB, Graef JD, et al. Transcriptome analyses reveal protein and domain families that delineate stage-related development in the economically important parasitic nematodes, *Ostertagia ostertagi* and *Cooperia oncophora*. BMC Genomics. 2013;14:118. 23432754 10.1186/1471-2164-14-118 PMC3599158

[15] Liu W, Tang H, Abuzeid AMI, Tan L, Wang A, Wang X, et al. Protein phosphorylation networks in spargana of *Spirometra erinaceieuropaei* revealed by phosphoproteomic analysis. Parasit Vectors. 2020;13:248. 32404185 10.1186/s13071-020-04119-w PMC7218563

[16] Liu SN, Su XY, Chen WQ, Yu JW, Li JR, Jiang P, et al. Transcriptome profiling of plerocercoid and adult developmental stages of the neglected medical tapeworm *Spirometra erinaceieuropaei*. Acta Trop. 2022;232:106483. 35469749 10.1016/j.actatropica.2022.106483

[17] Wang RJ, Li W, Liu SN, Wang SY, Jiang P, Wang ZQ, et al. Integrated transcriptomic and proteomic analyses of plerocercoid and adult *Spirometra mansoni* reveal potential important pathways in the development of the medical tapeworm. Parasit Vectors. 2023;16:316. 37670335 10.1186/s13071-023-05941-8 PMC10481575

[18] Li CY, Pan HZ, Liu W, Jin GH, Liu WZ, Liang CY, et al. Discovery of novel serum biomarkers for diagnosing and predicting postmenopausal osteoporosis patients by 4D-label free protein omics. J Orthop Res. 2023;41:2713–20. 37203779 10.1002/jor.25628

[19] Liu SN, Gao F, Wang RJ, Li W, Wang SY, Zhang X. Molecular characteristics of the fatty-acid binding protein (FABP) family in *Spirometra mansoni*—a neglected medical tapeworm. Animals (Basel). 2023;13:2855. 37760255 10.3390/ani13182855 PMC10525997

[20] Ren L, Li C, Shao W, Li C, Shao W, Lin W, et al. TiO2 with tandem fractionation (TAFT): an approach for rapid, deep, reproducible, and high-throughput phosphoproteome analysis. J Proteome Res. 2017;17:710–21. 29116813 10.1021/acs.jproteome.7b00520

[21] Capriotti AL, Cavaliere C, Ferraris F, Gianotti V, Laus M, Piovesana S, et al. New Ti-IMAC magnetic polymeric nanoparticles for phosphopeptide enrichment from complex real samples. Talanta. 2018;178:274–81. 29136822 10.1016/j.talanta.2017.09.010

[22] Cheng A, Grant CE, Noble WS, Bailey TL. MoMo: discovery of statistically significant post-translational modification motifs. Bioinformatics. 2019;35:2774–82. 30596994 10.1093/bioinformatics/bty1058 PMC6691336

[23] Conesa A, Götz S, García-Gómez JM, Terol J, Talón M, Robles M. Blast2GO: a universal tool for annotation, visualization and analysis in functional genomics research. Bioinformatics. 2005;21:3674–6. 16081474 10.1093/bioinformatics/bti610

[24] Harris MA, Clark J, Ireland A, Lomax J, Ashburner M, Foulger R, et al. The Gene Ontology (GO) database and informatics resource. Nucleic Acids Res. 2004;32:D258–61. 14681407 10.1093/nar/gkh036 PMC308770

[25] Maere S, Heymans K, Kuiper M. BiNGO: a Cytoscape plugin to assess overrepresentation of gene ontology categories in biological networks. Bioinformatics. 2005;21:3448–9. 15972284 10.1093/bioinformatics/bti551

[26] Shannon P, Markiel A, Ozier O, Baliga NS, Wang JT, Ramage D, et al. Cytoscape: a software environment for integrated models of biomolecular interaction networks. Genome Res. 2003;13:2498–504. 14597658 10.1101/gr.1239303 PMC403769

[27] Hornbeck PV, Kornhauser JM, Tkachev S, Zhang B, Skrzypek E, Murray B, et al. PhosphoSitePlus: a comprehensive resource for investigating the structure and function of experimentally determined post-translational modifications in man and mouse. Nucleic Acids Res. 2012;40:D261–70. 22135298 10.1093/nar/gkr1122 PMC3245126

[28] Szklarczyk D, Franceschini A, Wyder S, Forslund K, Heller D, Huerta-Cepas J, et al. STRING v10: protein-protein interaction networks, integrated over the tree of life. Nucleic Acids Res. 2015;43:D447–52. 25352553 10.1093/nar/gku1003 PMC4383874

[29] Chen T, Ma J, Liu Y, Chen ZG, Xiao N, Lu YT, et al. iProX in 2021: connecting proteomics data sharing with big data. Nucleic Acids Res. 2022;50:D1522–7. 34871441 10.1093/nar/gkab1081 PMC8728291

[30] Kim DW, Yoo WG, Lee M-R, Yang HW, Kim YJ, Cho SH, et al. Transcriptome sequencing and analysis of the zoonotic parasite *Spirometra erinacei* spargana (plerocercoids). Parasit Vectors. 2014;7:368. 25128015 10.1186/1756-3305-7-368 PMC4262225

[31] Ekka R, Gupta A, Bhatnagar S, Malhotra P, Sharma P. Phosphorylation of rhoptry protein RhopH3 is critical for host cell invasion by the malaria parasite. MBio. 2020;11:e00166-20. 33024030 10.1128/mBio.00166-20 PMC7542355

[32] Yang JC, Yang XK, Liu AQ, Li YQ, Niu ZP, Lyu CC, et al. The beta subunit of AMP-activated protein kinase is critical for cell cycle progression and parasite development in *Toxoplasma gondii*. Cell Mol Life Sci. 2022;79:532. 36205781 10.1007/s00018-022-04556-z PMC11802946

[33] Tian M, Chen X, Xiong Q, Xiong J, Xiao C, Ge F, et al. Phosphoproteomic analysis of protein phosphorylation networks in *Tetrahymena thermophila*, a model single-celled organism. Mol Cell Proteomics. 2014;13:503–19. 24200585 10.1074/mcp.M112.026575 PMC3916650

[34] An WF, Germain AR, Bishop JA, Nag PP, Metkar S, Ketterman J, et al. Discovery of potent and highly selective inhibitors of GSK3b. Bethesda MD: National Center for Biotechnology Information (US). 2010–2012. 23658955

[35] Simard E, Kovacs JJ, Miller WE, Kim J, Grandbois M, Lefkowitz RJ. β-arrestin regulation of myosin light chain phosphorylation promotes AT1aR-mediated cell contraction and migration. PLoS ONE. 2013;8:e80532. 24255721 10.1371/journal.pone.0080532 PMC3821855

[36] Henderson CA, Gomez CG, Novak SM, Lei MM, Gregorio CC. Overview of the muscle cytoskeleton. Compr Physiol. 2017;7:891–944. 28640448 10.1002/cphy.c160033 PMC5890934

[37] Linke WA. Titin gene and protein functions in passive and active muscle. Annu Rev Physiol. 2018;80:389–411. 29131758 10.1146/annurev-physiol-021317-121234

[38] Marat AL, Haucke V. Phosphatidylinositol 3-phosphates-at the interface between cell signalling and membrane traffic. EMBO J. 2016;35:561–79. 26888746 10.15252/embj.201593564 PMC4801949

[39] Hofmann F. The biology of cyclic GMP-dependent protein kinases. J Biol Chem. 2005;280:1–4. 15545263 10.1074/jbc.R400035200

[40] Nakamura T, Tsujita K. Current trends and future perspectives for heart failure treatment leveraging cGMP modifiers and the practical effector PKG. J Cardiol. 2021;78:261–8. 33814252 10.1016/j.jjcc.2021.03.004

[41] Hofmann F, Feil R, Kleppisch T, Schlossmann J. Function of cGMP-dependent protein kinases as revealed by gene deletion. Physiol Rev. 2006;86:1–23. 16371594 10.1152/physrev.00015.2005

[42] Fujiwara M, Hino T, Miyamoto R, Inada H, Mori I, Koga M, et al. The importance of cGMP signaling in sensory cilia for body size regulation in *Caenorhabditis elegans*. Genetics. 2015;201:1497–510. 26434723 10.1534/genetics.115.177543 PMC4676540

[43] Mery F, Belay AT, So AK, Sokolowski MB, Kawecki TJ. Natural polymorphism affecting learning and memory in *Drosophila*. Proc Natl Acad Sci U S A. 2007;104:13051–5. 17640898 10.1073/pnas.0702923104 PMC1941815

[44] Juang BT, Gu C, Starnes L, Palladino F, Goga A, Kennedy S, et al. Endogenous nuclear RNAi mediates behavioral adaptation to odor. Cell. 2013;154:1010–22. 23993094 10.1016/j.cell.2013.08.006 PMC4274153

[45] Liao K, Lv DY, Yu HL, Chen H, Luo SX. iNOS regulates activation of the NLRP3 inflammasome through the sGC/cGMP/PKG/TACE/TNF-α axis in response to cigarette smoke resulting in aortic endothelial pyroptosis and vascular dysfunction. Int Immunopharmacol. 2021;101:108334. 34768128 10.1016/j.intimp.2021.108334

[46] You JY, Liu XW, Bao YX, Shen ZN, Wang Q, He GY, et al. A novel phosphodiesterase 9A inhibitor LW33 protects against ischemic stroke through the cGMP/PKG/CREB pathway. Eur J Pharmacol. 2022;925:174987. 35490726 10.1016/j.ejphar.2022.174987

[47] Numata G, Takimoto E. Cyclic GMP and PKG signaling in heart failure. Front Pharmacol. 2022;13:792798. 35479330 10.3389/fphar.2022.792798 PMC9036358

[48] Hogan PG, Rao A. Dissecting ICRAC, a store-operated calcium current. Trends Biochem Sci. 2007;32:235–45. 17434311 10.1016/j.tibs.2007.03.009

[49] Cao Y, Du M, Luo S, Xia Y. Calcineurin modulates growth, stress tolerance, and virulence in *Metarhizium acridum* and its regulatory network. Appl Microbiol Biotechnol. 2014;98:8253–65. 24931310 10.1007/s00253-014-5876-3

[50] Ren HN, Liu RD, Song YY, Zhuo TX, Guo KX, Zhang Y, et al. Label-free quantitative proteomic analysis of molting-related proteins of *Trichinella spiralis* intestinal infective larvae. Vet Res. 2019;50:70. 31547875 10.1186/s13567-019-0689-0 PMC6757440

[51] Chai YN, Qin J, Li YL, Tong YL, Liu GH, Wang XR, et al. TMT proteomics analysis of intestinal tissue from patients of irritable bowel syndrome with diarrhea: implications for multiple nutrient ingestion abnormality. J Proteomics. 2021;231:103995. 33011346 10.1016/j.jprot.2020.103995

[52] Anirudhan A, Angulo-Bejarano PI, Paramasivam P, Manokaran K, Kamath SM, Murugesan R, et al. RPL6: a key molecule regulating zinc- and magnesium-bound metalloproteins of Parkinson’s disease. Front Neurosci. 2021;15:631892. 33790735 10.3389/fnins.2021.631892 PMC8006920

[53] Bai D, Zhang J, Xiao W, Zheng X. Regulation of the HDM2-p53 pathway by ribosomal protein L6 in response to ribosomal stress. Nucleic Acids Res. 2014;42:1799–811. 24174547 10.1093/nar/gkt971 PMC3919599

[54] Kenmochi N, Yoshihama M, Higa S, Tanaka T. The human ribosomal protein L6 gene in a critical region for Noonan syndrome. J Hum Genet. 2000;45:290–3. 11043511 10.1007/s100380070018

[55] Valencia-Hernandez AM, Ng WY, Ghazanfari N, Ghilas S, de Menezes MN, Holz LE, et al. A natural peptide antigen within the *Plasmodium* Ribosomal protein RPL6 confers liver TRM cell-mediated immunity against malaria in mice. Cell Host Microbe. 2020;27:950-962.e7. 32396839 10.1016/j.chom.2020.04.010

[56] Mager WH. Control of ribosomal protein gene expression. Biochim Biophys Acta. 1988;949:1–15. 3275463 10.1016/0167-4781(88)90048-6

[57] An Y, Wang Y, Xu G, Liao Y, Huang G, Jin X, et al. Identification of key genes in osteosarcoma - before and after CDK7 treatment. Medicine (Baltimore). 2021;100:e27304. 34596127 10.1097/MD.0000000000027304 PMC8483848

[58] Ma D, Liu P, Wen J, Gu Y, Yang Z, Lan J, et al. FCN3 inhibits the progression of hepatocellular carcinoma by suppressing SBDS-mediated blockade of the p53 pathway. Int J Biol Sci. 2023;19:362–76. 36632465 10.7150/ijbs.69784 PMC9830510

