# Supplementary material for: Comparative analysis of phosphorylated proteomes between plerocercoid and adult Spirometra mansoni reveals phosphoproteomic profiles of the medical tapeworm
Source: Parasit Vectors. 2024 Aug 31;17:371. doi: 10.1186/s13071-024-06454-8 (PMC11366163; doi:10.1186/s13071-024-06454-8)
Supplement: Supplementary file 5 — Supplementary Material 5. Table S5. Primer sequences designed for RT-qPCR. Figure S1. Peptide length of all identified proteins. Figure S2. Pie chart of the number distribution of phosphorylation sites on identified phosphorylated peptide segments. Figure S3. GO secondary classification chart. Figure S4. Functional classification of COG. Figure S5. KEGG annotation statistics. Figure S6. Differential protein GO annotation bar chart. Figure S7. KEGG enrichment bubble chart. Figure S8. Ipath integration path diagram of metabolic pathway map. Figure S9. Ipath integration path diagram of microbial metabolic pathway map. Figure S10. Ipath integration path diagram of secondary metabolic pathway map. [file 13071_2024_6454_MOESM5_ESM.docx]

**Supplementary materials**

**Comparative analysis of phosphorylated proteomes between plerocercoid and adult *Spirometra mansoni* reveals phosphoproteomic profiles of the medical tapeworm**

**Table S5.** Primers sequences designed for RT-qPCR.

**Figure S1.** Peptide length of all identified proteins.

**Figure S2.** Pie chart of the number distribution of phosphorylation sites on identified phosphorylated peptide segments.

**Figure S3.** GO secondary classification chart.

**Figure S4.** Functional classification of COG.

**Figure S5.** KEGG annotation statistics.

**Figure S6.** Differential protein GO annotation bar chart.

**Figure S7.** KEGG enrichment bubble chart.

**Figure S8.** Ipath integration path diagram of metabolic pathway map.

**Figure S9.** Ipath integration path diagram of microbial metabolic pathway map.

**Figure S10.** Ipath integration path diagram of secondary metabolic pathway map.

**Table S5.** Primers sequences designed for RT-qPCR.

| **Stage** | **Protein description** | **Accession** | **Primer Sequence (5' to 3')** | **Product size (bp)** |
| --- | --- | --- | --- | --- |
| Adult | Nucleoprotein TPR | A0A7M3PRG3-S595 | F-TGCTGGCGGCGTATCTTT  R-GTTCCTGCTGCTGCTGTG | 401 |
|  | Polynucleotide adenylyltransferase | A0A7M3PRQ6-S607 | F-TTCCTCGGTGGTGTTAGTT  R-AAGAACCCTCAGTCTGGAT | 477 |
|  | Ribosome assembly factor mrt4 | A0A7M3PS07-S258 | 1. TGTGCCCTCCTCTTCTCC   R-GAGTCCCAACGAGCCAAT | 332 |
|  | UV excision repair protein RAD23 | A0A7M3PS32-S281 | F-CGCCAGCCACAACATCTC  R-GGCTTCAACCGTGGGAAT | 212 |
|  | 1. acyltransferase | A0A7M3PSR3-S11 | F-ACTTTCTATTTGCTCCTACCC  R-TTTAGCGGAACTGAGACC | 445 |
|  | Eukaryotic translation initiation factor 5A | A0A7M3QUY0-S2 | F-ATCGTCGATGCCTCTTCC  R-GTGGTGCCGTTGTTTAGT | 176 |
| plerocercoid | 40S ribosomal protein S3a | A0A7M3Q1R5-S243 | F-TTCACGAAACGGAACTGT  R-TCCTCGACCTGCAATCTA | 158 |
|  | N-acetyl-D-glucosamine kinase | A0A7M3QF48-S74 | F-TGCCGCAACCGATAAAGC  R-TTCGTCGTCATCCACAAAGAGT | 178 |
|  | Paramyosin | A0A7M3Q648-S483 | F-GCCTGGATGAACTGAGTGG  R-GGTCGTCGGTAAGACCCT | 351 |
|  | Phosphotransferase | A0A7M3QVA0-S245 | F-GGCAAACTAGAGCCACCT  R-ATTCCGTCCGACAACACC | 255 |
|  | Phosphopyruvate hydratase | A0A7M3PSD4-S409 | F-ATGAAGCCGTGGAGTTGC  R-GAGGGAGACGCCGAGAAT | 218 |
|  | Hypothetical protein | A0A7M3PXT0-S589 | F-TCAGACTCCCGCCTCCAT  R-ACGGCAAGCATCAGTCCTC | 309 |
|  | Hypothetical protein | A0A7M3Q4P3-S94 | F-AAGCAATCGGAGACATCAGT  R-GGAGATGGCTTCTACACCC | 101 |
|  | Hypothetical protein | A0A7M3Q9S7-S121 | F-AGACTCCCGAAGTATCAGC  R-ACACTTTCTTCCCGCTCT | 150 |
|  | Hypothetical protein | A0A7M3QB76-S6 | F-TAGTCCTGCCTCGGTCAT  R-GCTGCTGGTATGCGTAGAT | 374 |
|  | Hypothetical protein | A0A7M3RFA9-S240 | F-ATCGTGCTGGACTCGGGTGA  R-AGGCGGCAGTAGCCATCTCC | 244 |
| Reference gene | glyceraldehyde-phosphatedehydrogenase (GADPH) |  | F-AGCAACCTCGTTGATGTCGT  R-TGAATTGACCGTGGGTGGAG | 97 |


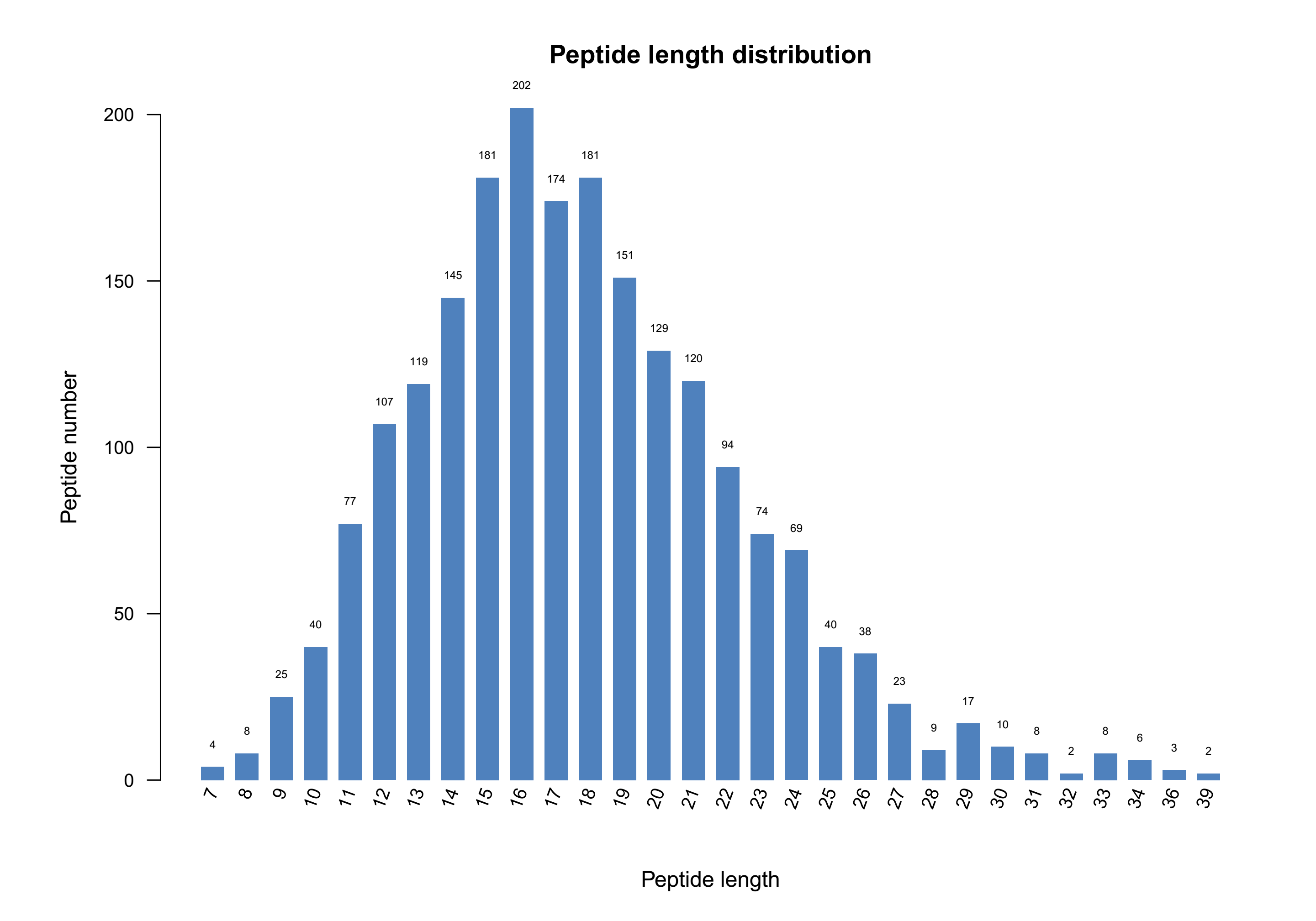
**Figure S1.** Peptide length of all the identified proteins.


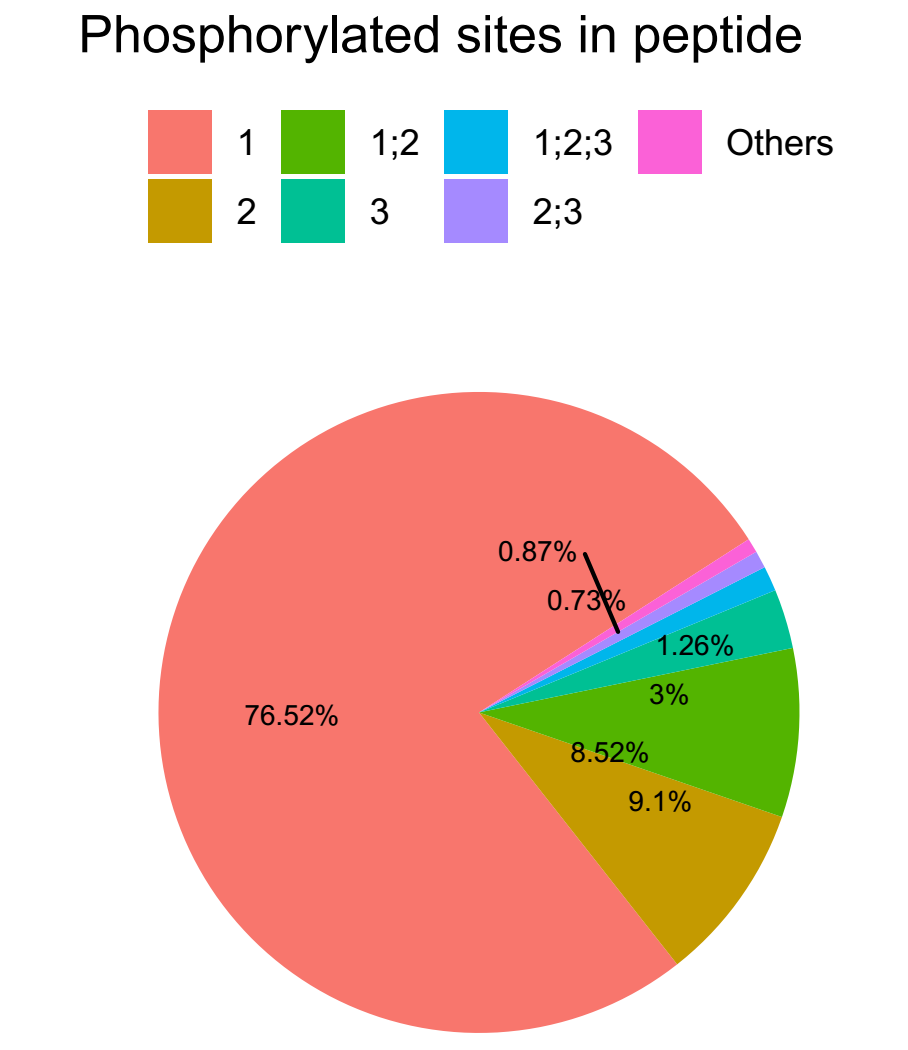


**Figure S2.** Pie chart of the number distribution of phosphorylation sites on identified phosphorylated peptide segments. Red indicates only one phosphorylation site on the peptide, green indicates two phosphorylation sites, and blue indicates three phosphorylation sites.


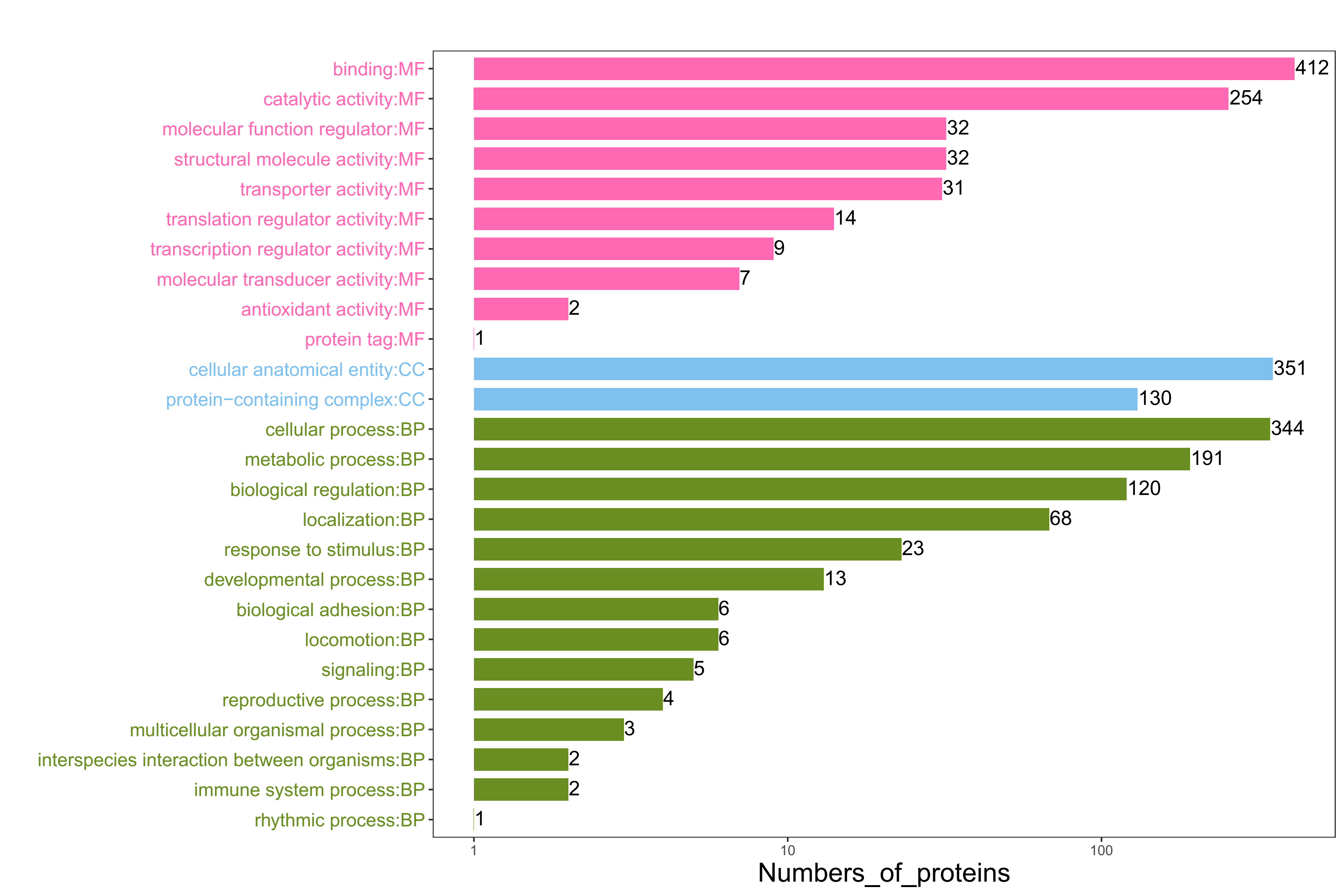
**Figure S3.** GO secondary classification. BP (biological process) green, CC (cellular component) blue, MF (molecular function) red.


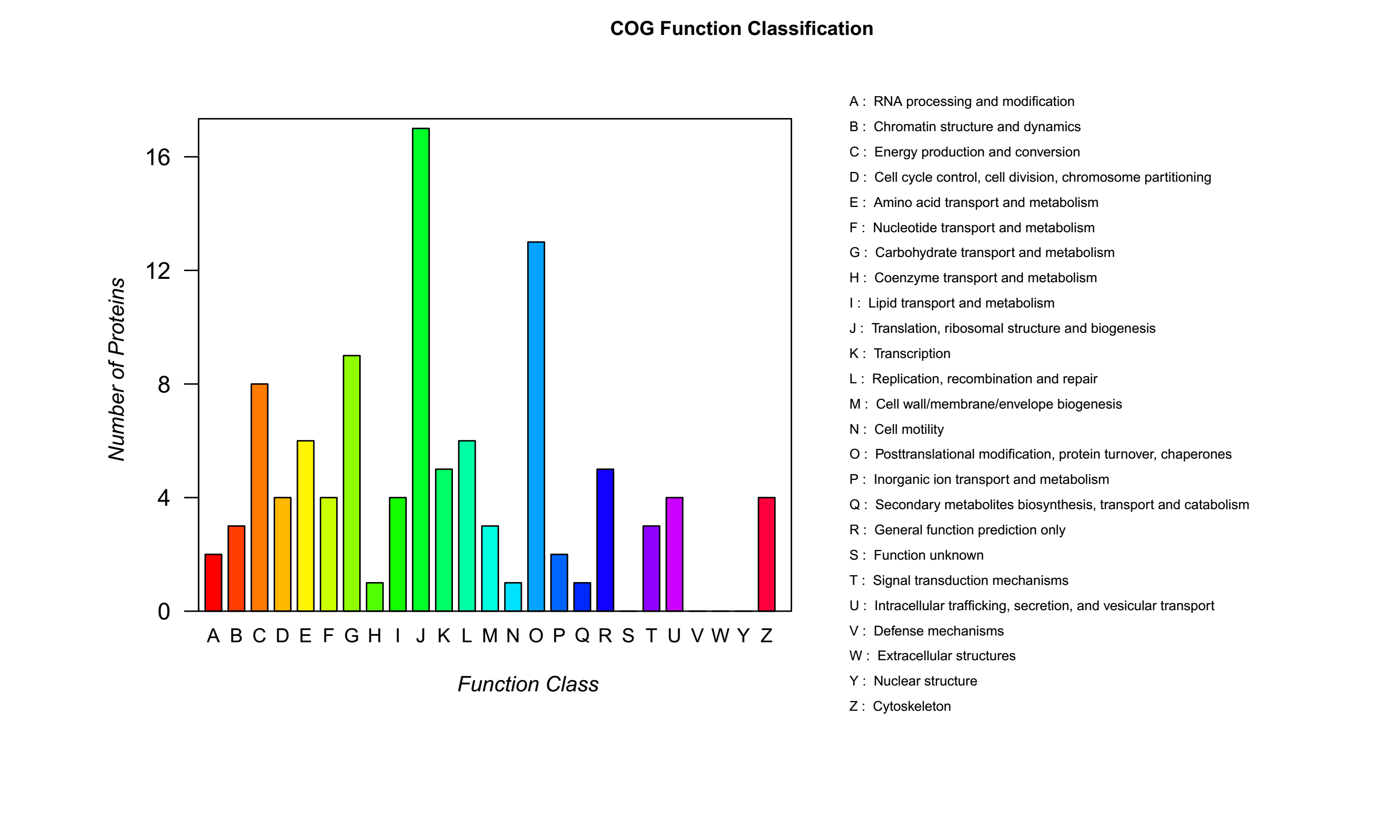
**Figure S4.** Functional classification of COG. The X-axis represents classifcation, while the Y-axis represents the number of identifed proteins.


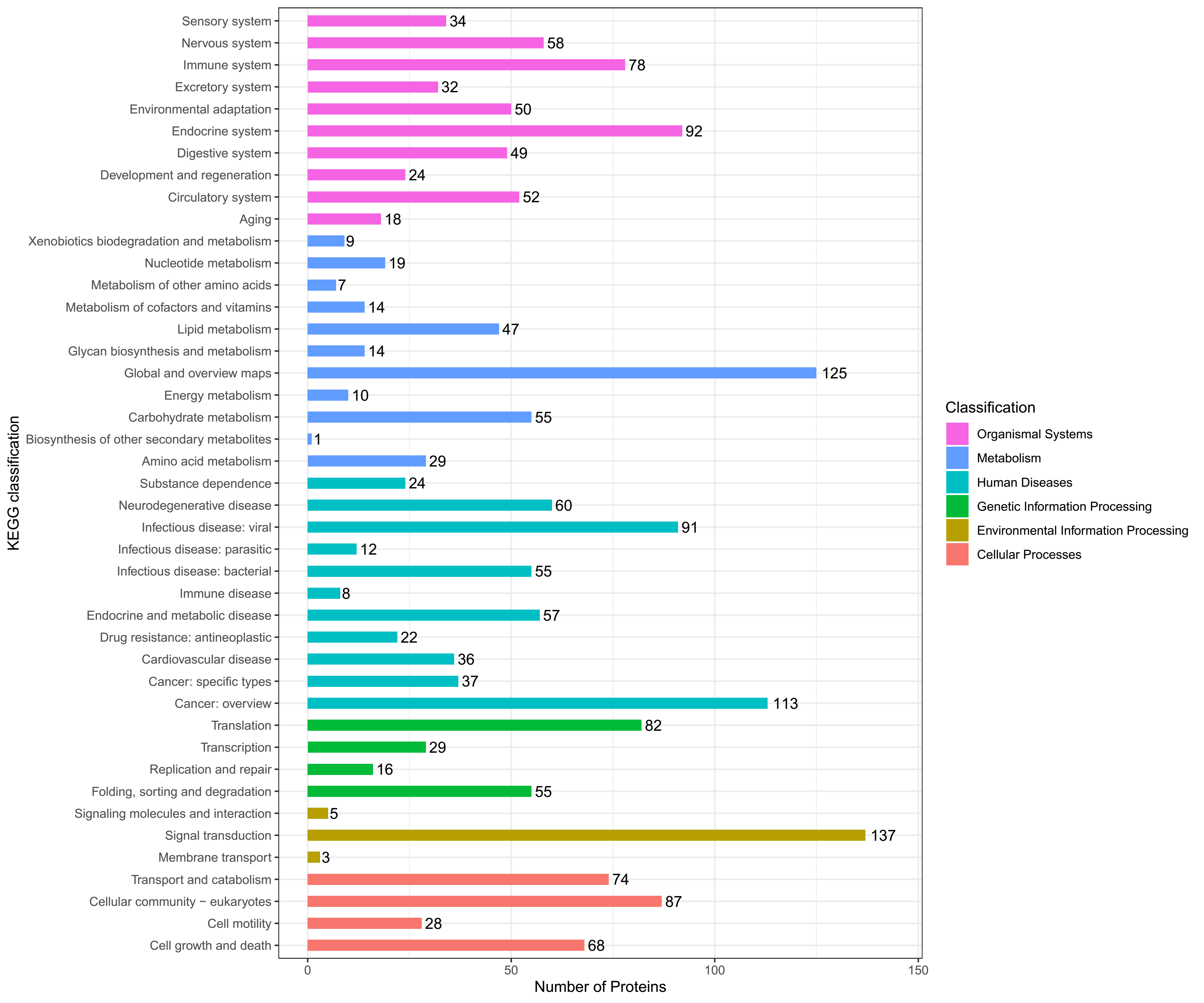


**Figure S5.** KEGG annotation statistics. Note: The ordinate is the name of KEGG metabolic pathway, and the abscissa is the number of proteins annotated to this pathway.

**
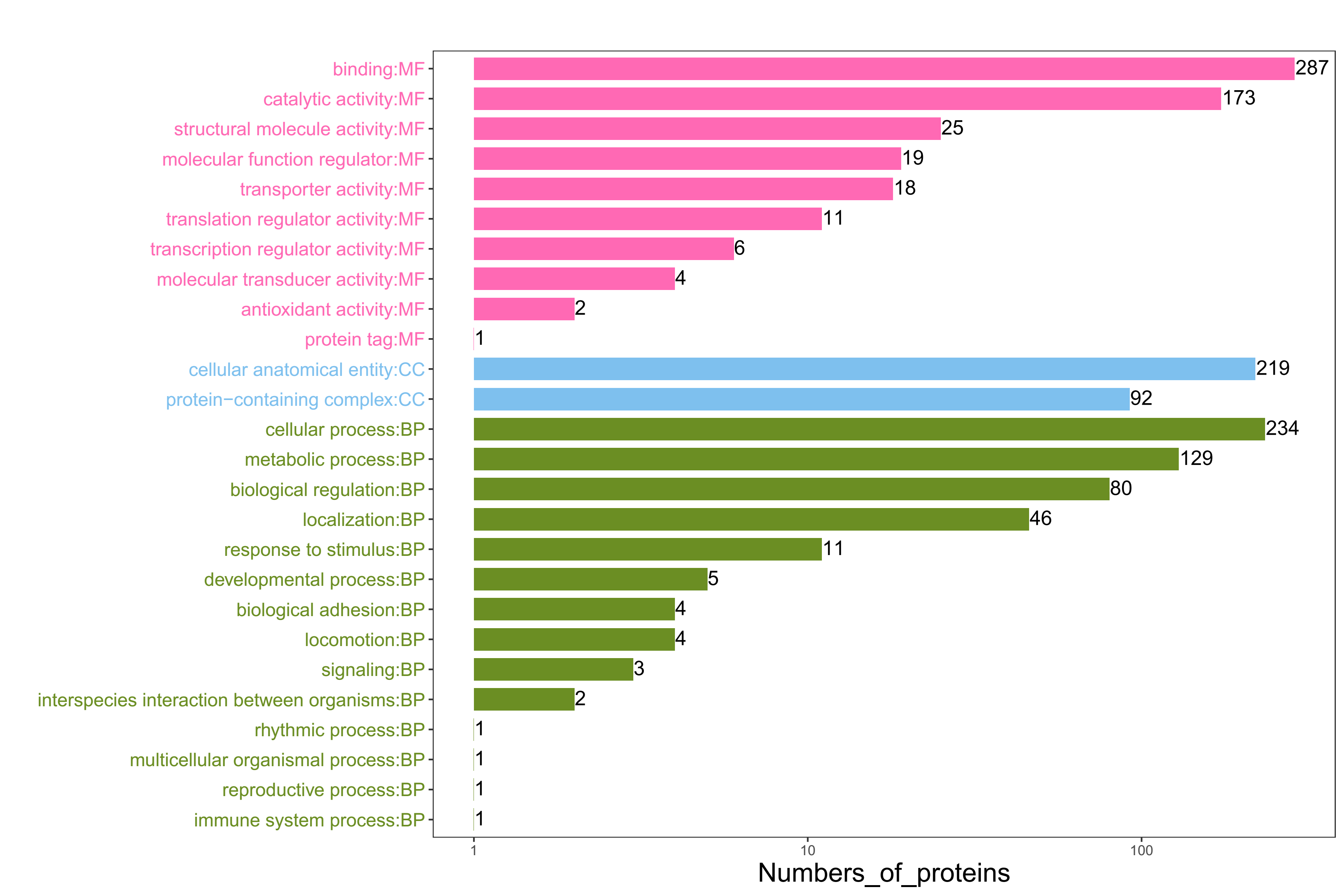
**

**Figure S6.** Differential protein GO annotation bar chart. BP (biological processes) green, CC (cell components) blue, MF (molecular functions) red.


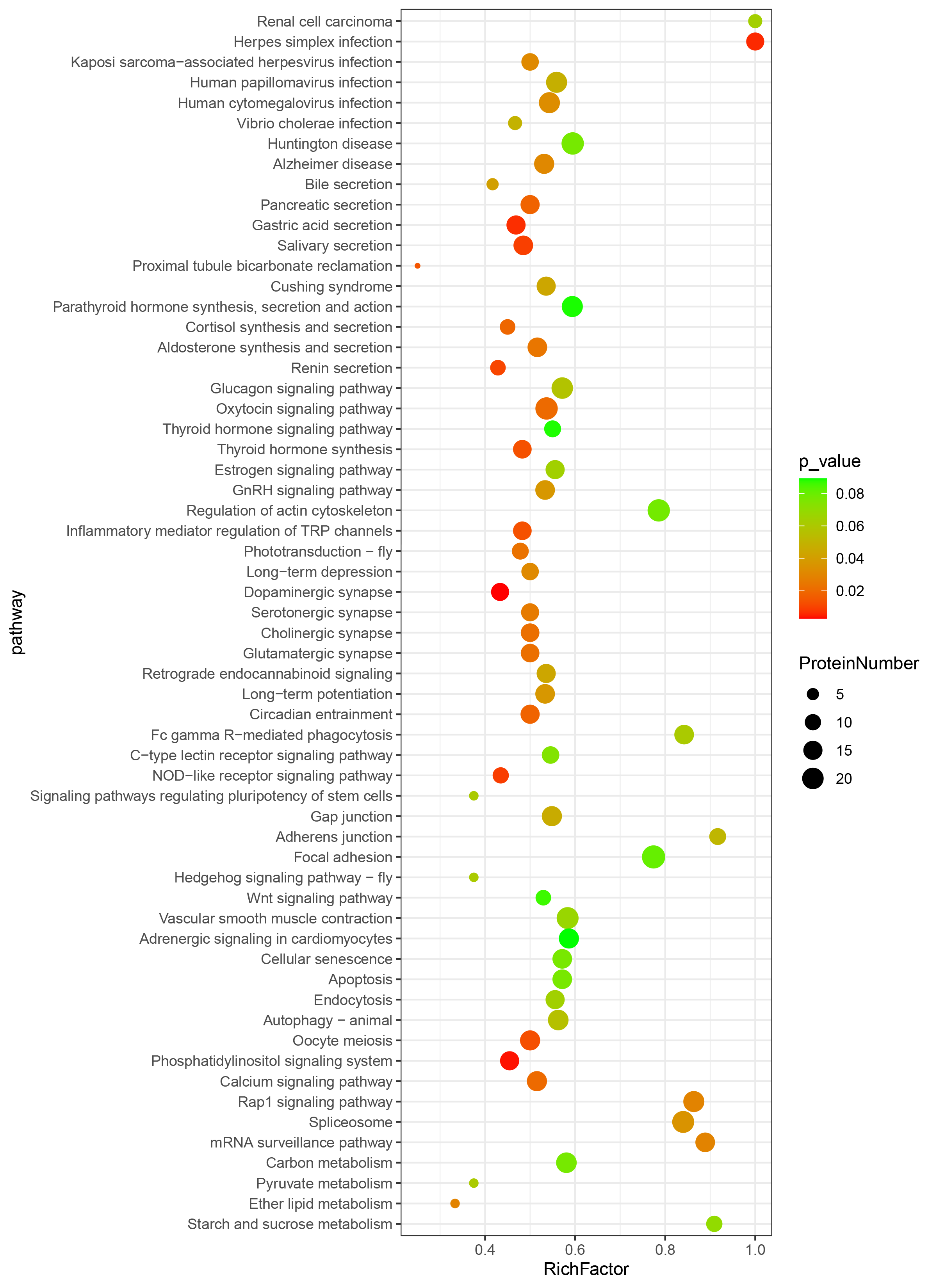


**Figure S7.** KEGG enrichment bubble chart (CC vs YC). KEGG enrichment analysis of differentially expressed proteins in adults vs. plerocercoids.

**
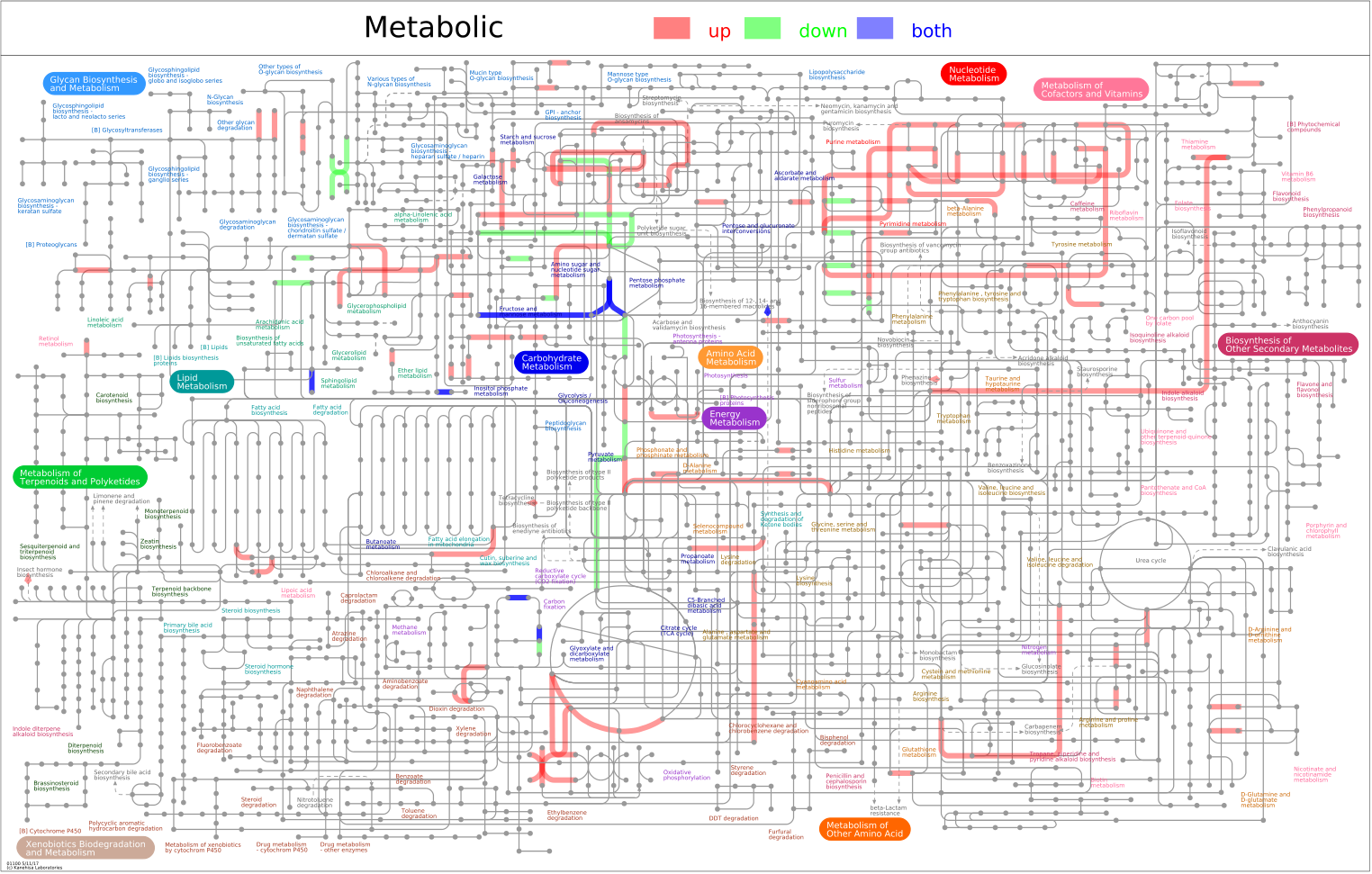
**

**Figure S8.** Ipath integration path diagram of metabolic pathway map. Note: The nodes in the figure represent different compounds, and the boundaries represent different enzymatic reactions. The red lines represent reactions involving up-regulated proteins, the green lines represent reactions involving down-regulated proteins, and the blue lines represent reactions involving both up-regulated and down-regulated proteins. Different lines represent different types of metabolic pathways or functions.

**
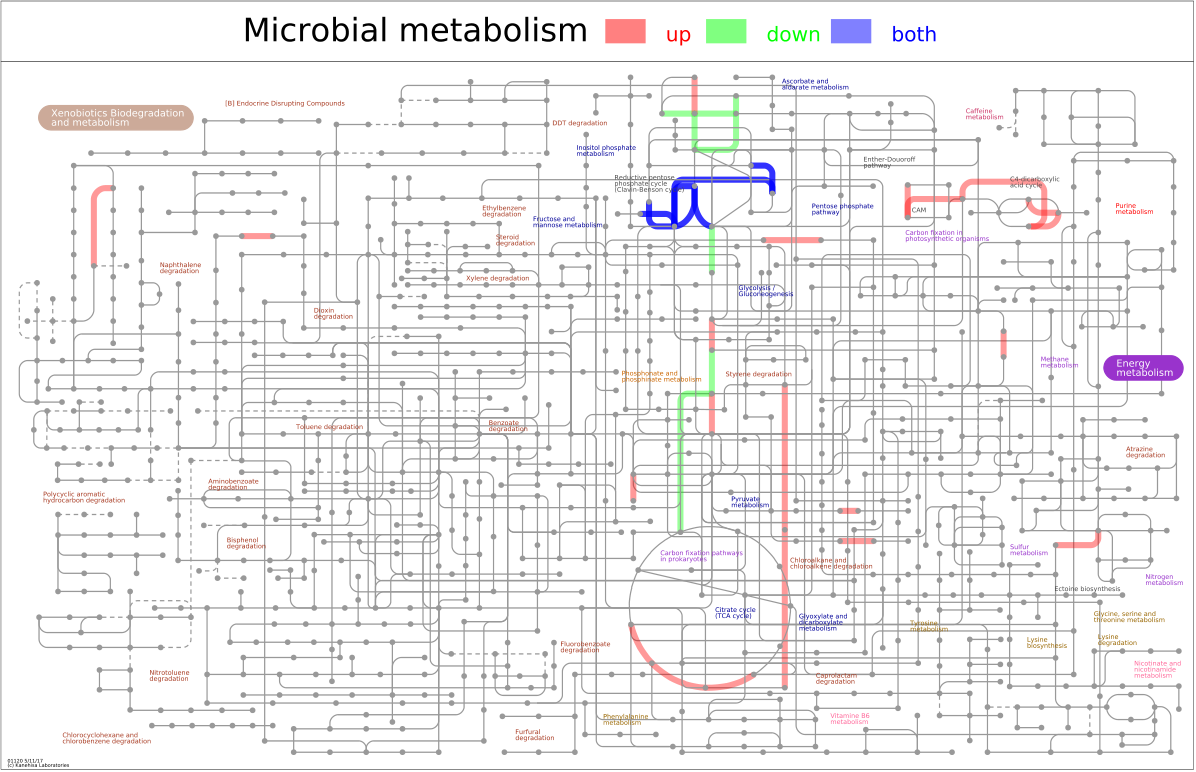
**

**Figure S9.** Ipath integration path diagram of microbial metabolic pathway map.

**
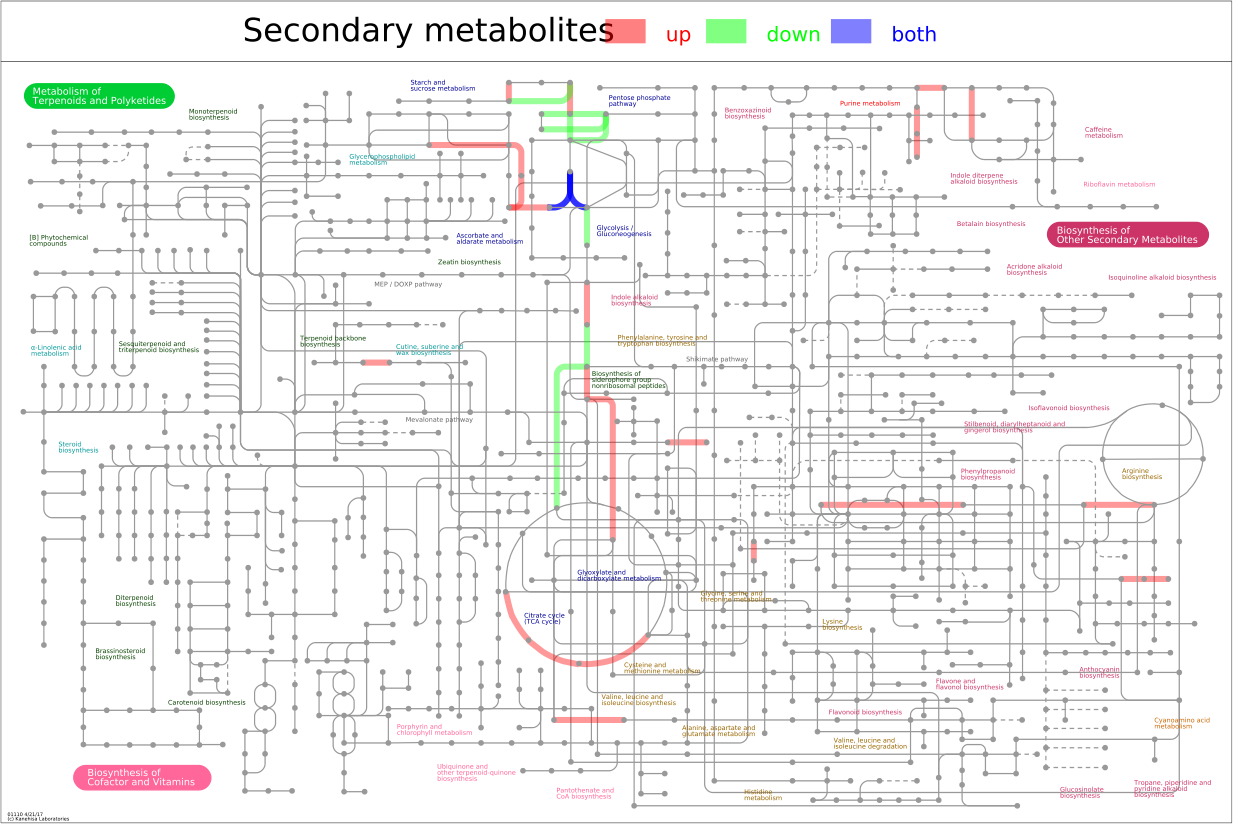
**

**Figure S10.** Ipath integration path diagram of secondary metabolic pathway map.
